# Cross-crisis effects of COVID-19 on air pollution risk perception and mental stress

**DOI:** 10.1038/s41598-026-53900-x

**Published:** 2026-06-02

**Authors:** Yuxin Liu, Jingjing Bi, Chen Li, Chengyu Fan, Sijia Zhou, Lei Huang

**Affiliations:** 1 State Key Laboratory of Water Pollution Control and Green Resource Recycling, School of the Environment, Nanjing University, Nanjing, 210023 China; 2 Teaching Department of Basic Courses, Jiangsu Vocational Institute of Commerce, Nanjing, 211168 China; 3 Center for Public Health Research, Medical School, Nanjing University, Nanjing, 210093 China; 4 Department of Information Engineering, Meizhouwan Vocational Technology College, Putian, 351119 China; 5 Basic Science Center for Energy and Climate Change, Beijing, 100081 China

**Keywords:** Air pollution, COVID-19 pandemic, Cross-crisis effects, Mental stress, Risk perception, Environmental social sciences, Psychology, Psychology, Risk factors

## Abstract

Against the backdrop of a rapidly evolving multi-crisis era, the COVID-19 pandemic raises critical questions about how acute shocks may reconfigure perceptions of persistent environmental risks. Drawing on two representative surveys in Nanjing before (2018–2019, n = 508) and during (2022, n = 500) the pandemic, triangulated with geotagged Nanjing Weibo posts (2019–2022) and the World Risk Poll (2019; 2021), this study develops three theoretical models and tests eight hypotheses. Results reveal clear cross-crisis effects: COVID-19 significantly reduced perceived air-pollution risks, consistent with a finite-pool-of-worry account. This decline partially offset stress, yet the pandemic still produced a net increase in mental stress by introducing additional psychological burdens. Although COVID-19 reduced average daily PM_2.5_ exposure and increased capacity-based trust in air-pollution governance, neither mediated changes in risk perception. Crucially, the crisis amplified the negative impact of perceived physical symptoms on mental well-being, heightening vulnerability under crisis conditions. These findings show how acute crises spill over to reshape chronic risk perception and psychological consequences. They underscore the importance of integrating environmental governance with compound risk communication and timely mental health support. The conclusions are transferable to other compound crises, including future pandemics, extreme weather events, or environmental emergencies, offering valuable lessons for strengthening societal resilience.

**Supplementary Information:**

The online version contains supplementary material available at 10.1038/s41598-026-53900-x.

## Introduction

In the past decade, societies worldwide have been reshaped by intensifying, overlapping environmental and societal crises. Extreme weather events, energy disruptions, and recurrent public-health emergencies have created a landscape in which people increasingly appraise risks not in isolation but in relation to other concurrent threats^1^. The COVID-19 pandemic represents one of the most salient acute shocks within this multi-crisis era, rapidly reshaping social functioning, governance practices, and public psychological states—while also, unexpectedly, improving air quality in many cities. Yet it remains unclear whether such an abrupt, large-scale crisis can redefine how people understand long-standing environmental risks^2^. When an acute health threat dominates public attention, does concern for chronic hazards such as air pollution diminish, intensify, or undergo a more fundamental reconfiguration? Despite their importance for environmental governance and risk communication, these questions remain largely unanswered.

Air pollution remains one of the most pressing environmental and public health challenges in China. Elevated levels of fine particulate matter (PM_2.5_) and other pollutants contribute are strongly associated with premature mortality, respiratory and cardiovascular diseases, and substantial economic burdens^3^. Although nationwide initiatives such as the Air Pollution Prevention and Control Action Plan and the Blue Sky Protection Campaign have yielded notable improvements over the past decade, polluted air persists as a chronic and highly salient hazard^4,5^. Individuals encounter it daily through visible haze, respiratory discomfort, and intensive media attention. Beyond shaping behavioral compliance and policy support, risk perception is increasingly recognized in environmental psychology as a critical mediator linking environmental hazards to psychological well-being^6^. Perceiving air pollution as a serious health threat can, in itself, exacerbate mental stress^7^, underscoring the importance of understanding how such perceptions evolve when society is disrupted by broader crises. These features position air pollution as an especially suitable context for investigating how a major societal crisis may reconfigure perceptions of a long-standing environmental threat.

In stark contrast, the COVID-19 pandemic epitomizes an acute, large-scale crisis. Spreading rapidly across continents in early 2020, it generated cascading effects far beyond direct morbidity and mortality^8^. Severe economic recessions, rising poverty, and widening social inequality followed in its wake^9,10^. Such disruptions underscore a defining feature of global crises: their impacts transcend specific domains, reverberating across society and spilling over into broader perceptions of environmental risks^1,11,12^. Epidemiologists and crisis scholars caution that emergencies of this nature are likely to recur^13,14^, highlighting the urgency of examining how acute crises reconfigure public risk perceptions and reshape relations between citizens and institutions. The pandemic therefore offers a rare opportunity to explore how an acute shock may spill over into broader systems of risk perception. At the same time, whether such spillovers are context-specific or generalizable remains uncertain; globally comparable evidence is needed to assess whether crisis-driven reallocation of concern extends beyond a single city or country.

While research on COVID-19 has illuminated its direct consequences—mobility restrictions, diminished social interaction, and heightened psychological distress^15,16^.—its implications for perceptions of environmental risks remain underexplored. As a sudden external shock, the pandemic provides a valuable setting for examining linkages between otherwise weakly related risks. In previous research on the effects of COVID-19 on other risks, two competing perspectives dominate theoretical debates^11^. The finite pool of worry hypothesis contends that acute survival threats monopolize attention, suppressing concern for other risks^17^. Conversely, the affect generalization hypothesis argues that crises intensify a generalized sense of vulnerability, amplifying worry across domains^18^. Climate change has served as the primary empirical testbed, yet evidence on the relationship between COVID-19 and air-pollution risk perception remains limited, particularly in China^18^. However, climate change is relatively abstract and psychologically distant. Because it is not directly encountered in everyday life, attention to it may be especially vulnerable to displacement during an acute crisis. Air pollution offers a different test. Like climate change, it is chronic; unlike climate change, it is visible, routinized and tied to bodily symptoms. A critical question thus arises: when a new crisis dominates public attention, does it suppress concern for enduring threats such as polluted air, or does it heighten awareness of vulnerability? Examining whether COVID-19 reshaped air-pollution risk perception therefore not only provides a stronger test of how an acute shock reorders concern for a chronic, everyday hazard, but also extends cross-crisis research beyond abstract environmental threats by exploring the relationship between two everyday risks, thereby enriching theories of cross-crisis interaction. Moreover, because both finite pool of worry and affect generalization hypothesis work through attention and salience, combining surveys with social-media attention indicators offers a more direct test of whether COVID-19 crowded out concern about chronic environmental risks.

Potential mechanisms further complicate this picture. We examine two possible pathways through which the COVID-19 crisis may have shaped perceptions of air pollution risks. On the one hand, changes in objective exposure could shape perceptions. During the pandemic, industrial shutdowns and mobility restrictions substantially reduced air pollutant concentrations in many Chinese cities^19^. Such visible improvements—for example, clearer skies and fewer haze days—likely provided salient cues that reinforced perceptions of reduced health risks. On the other hand, crisis governance may influence perceptions through capacity-based trust in government. Effective government responses, including timely information disclosure and decisive containment measures, demonstrate state capacity and can strengthen confidence beyond the immediate crisis domain^20,21^. This potential “trust spillover” suggests that trust gained during pandemic management might extend to the domain of air-pollution governance. Air pollution is especially well suited to testing these pathways. Unlike climate change, it combines measurable exposure with visible cues and embodied discomfort, making it possible to contrast objective exposure with subjective experience. In China, moreover, air pollution has long been a prominent governance issue, providing a useful setting in which to examine whether trust gained during a health emergency spills over into environmental risk perception. Taken together, these two mediating pathways—objective exposure and capacity-based trust in government—have been widely theorized but remain underexplored in the context of concurrent crises, highlighting the need for empirical testing. Unpacking how exposure shifts and trust transfer jointly shape risk perception is therefore essential for understanding crisis-driven change in environmental attitudes.

The implications extend directly to public mental health. Pandemic control measures disrupted both industrial production and everyday life, and COVID-19 itself has been shown to significantly elevate mental stress worldwide^22–24^. Yet the role of the COVID-19 crisis in shaping the relationship between air pollution risk perception and mental stress remains unclear. We explore two potential pathways through which the pandemic may influence stress: first, by increasing or reducing risk perception of air pollution; and second, by moderating the effect of self-perceived pollution-related physical symptoms on stress. Risk perceptions of air pollution are positively associated with mental stress and anxiety^25^. This raises a critical question: by reshaping perceptions of air pollution, did the pandemic indirectly exacerbate or mitigate mental stress? Addressing this question allows us to move beyond the direct psychological impacts of crises and uncover the indirect pathways through which changes in environmental risk perceptions shape mental health. Understanding how crisis-induced changes in risk perception relate to psychological stress is thus crucial for assessing resilience under multi-crisis conditions.

Leveraging a quasi-natural experiment created by COVID-19 control measures, we integrate three complementary data sources to examine how an acute public-health shock reshaped perceptions of a chronic environmental risk and its mental-health implications. We combine two comparable survey waves in Nanjing (2018–2019; 2022), geotagged Sina Weibo posts from Nanjing (2019–2022) tracking quarterly attention to COVID-19 and air pollution, and the World Risk Poll (2019; 2021) capturing cross-national shifts in perceived “greatest daily-life threats” across income contexts. We ask whether COVID-19 crowded out concern about air pollution, through which pathways (exposure change vs. trust transfer), and how these shifts reconfigured mental stress. Drawing on this triangulated evidence, the study advances theorizing on cross-crisis spillover and underscores the need for compound risk communication that integrates environmental and mental-health concerns to strengthen resilience to future shocks. As large-scale crises such as pandemics, extreme weather events, and energy disruptions are likely to recur, our findings offer timely insights for navigating the complexities of compound risks.

### Theoretical models and hypotheses

**Fig. 1:**
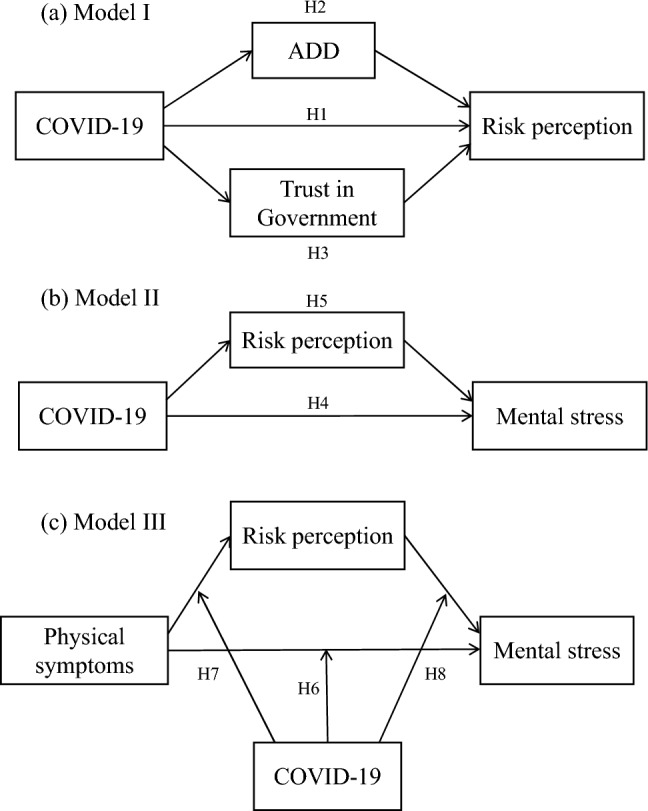
Theoretical models and hypotheses.

As illustrated in Fig. 1, we develop three theoretical models and eight hypotheses to examine how the COVID-19 pandemic shaped public perceptions of air pollution risks and mental stress, explicitly treating the pandemic—pollution nexus as a cross-crisis system in which perception, affect and capacity-based trust in government can spill over across domains.

#### Theoretical perspectives on cross-crisis risk perception

In complex social settings, people often confront more than one risk at a time. Existing accounts of cross-crisis risk perception can be broadly distinguished by whether the risks are directly related. For directly related risks, one common situation involves two comparable risks that are evaluated within a shared decision space. For example, trade-offs between the risks of a disease and those of vaccination are often interpreted through the lens of secondary-risk theory^26^, while trade-offs between climate change and nuclear power are commonly discussed in terms of risk trade-off theory^27^. Another situation concerns the relationship between everyday, readily experienced risks and more abstract risks. Based on the availability heuristic, people tend to judge abstract risks through risks that are easier to recall or more directly experienced^28^. For instance, everyday exposure to air pollution ^29^ or experiences of extreme weather^30^ may shape perceptions of the more abstract risk of climate change. By contrast, for two relatively unrelated risks, existing research has focused mainly on two competing hypotheses: the finite-pool-of-worry hypothesis and the affect-generalization hypothesis. External shocks such as financial crises^31^ and COVID-19^18^ may crowd out concern about climate change, whereas negative experiences during the pandemic may also spill over and influence how the public perceives other risks^2^. Given the abstract nature of climate change, examining the relationship between COVID-19 and air-pollution risk perception can enrich theorizing about how an acute crisis becomes linked to a chronic, everyday hazard in public perception.

#### Model I: COVID-19 crisis and air pollution risk perception

First, regarding the cross-crisis effects of COVID-19 on air pollution risk perception. Crisis events not only generate immediate threats but also reshape public concern for other risks^1^. The finite-pool-of-worry hypothesis suggests that individuals possess limited cognitive and emotional resources; therefore, heightened anxiety about one acute threat may suppress concern to other issues^17^. In addition, rational prioritization refers to the process by which individuals subjectively compare and weigh multiple risks under conditions of concurrent threat^32,33^. Under acute survival pressure, individuals may also engage in a more deliberate reprioritization of risks, temporarily assigning lower priority and greater acceptability to chronic environmental hazards such as air pollution. In contrast, the affect-generalization hypothesis emphasizes that worry may spill over and amplify concerns across multiple domains^34^. Furthermore, risk society theory suggests that experiencing one risk may heighten broader risk awareness, rather than depleting cognitive resources^35^. Although prior evidence remains mixed, these perspectives provide competing predictions for how the pandemic might influence perceptions of chronic risks such as air pollution.

##### **Hypothesis 1a**

*The COVID-19 pandemic directly reduced public perception of air pollution risks.*

##### **Hypothesis 1b**

*The COVID-19 pandemic directly increased public perception of air pollution risks.*

Second, concerning the mediating role of objective exposure. Risk judgments are strongly shaped by personal experiences. Research shows that disasters and crises often lead individuals to interpret their experiences as signals of future hazards^36^. For air pollution—a risk routinely encountered in daily life—the availability heuristic offers a cognitive explanation: people evaluate risks based on vivid or easily retrievable examples, particularly those tied to strong emotions^37^. During the pandemic, reductions in industrial activity and mobility led to sharp declines in pollutant emissions, particularly in densely populated and industrialized regions such as Nanjing city^38^. These observable changes raise the possibility that reduced air pollution exposure during COVID-19 pandemic contributed to lower perceived health risks, thereby mediating the influence of COVID-19 on risk perception.

##### **Hypothesis 2**

*The COVID-19 pandemic indirectly reduced public perceptions of air pollution risks by lowering average daily exposure (ADD).*

Third, concerning the mediating role of trust in government’s capacity to control air pollution. According to the theory of governmental trustworthiness, effective crisis management—characterized by transparency, integrity, and responsiveness—strengthens public trust in government capacity^39,40^. During COVID-19, the Chinese government demonstrated decisive crisis management and rapid implementation of containment measures^41,42^. Building on the halo effect, citizens’ positive evaluations of government performance in one domain (e.g., epidemic control) are likely to spill over into perceptions of competence in other areas, such as environmental governance. Prior studies further suggest that higher levels of capacity-based trust in government often correlate with lower perceived risks^43^, as citizens place greater reliance on the state’s protective capacity. Thus, the pandemic may have reduced air pollution risk perception not only through exposure reduction but also by strengthening capacity-based trust in government.

##### **Hypothesis 3**

*The COVID-19 pandemic reduced public perceptions of air pollution risks by enhancing trust in government’s capacity to control air pollution.*

#### Model II: COVID-19 crisis and mental stress

Fourth, regarding the direct effect of crises on mental stress. Public health emergencies not only threaten physical safety but also disrupt mental well-being^44^. The acute stress induced by COVID-19, combined with large-scale lockdowns, social isolation, and economic uncertainty, has been shown to heighten risks of anxiety, depression, and trauma-related symptoms^45,46^. Accordingly, it is reasonable to expect that the pandemic directly increased public mental stress.

##### **Hypothesis 4**

*The COVID-19 pandemic directly heightened public mental stress.*

Fifth, concerning the mediating role of air pollution risk perception in the crisis–stress relationship. Environmental risk perceptions are closely linked to psychological well-being^47^. Lower concern about pollution may help relieve stress, whereas higher concern may exacerbate anxiety. Building on Hypotheses 1a and 1b, two pathways can be theorized. From a finite-pool-of-worry perspective, COVID-19 may shift concern away from air pollution, thereby lowering risk perception and alleviating stress. By contrast, from an affect-generalization perspective, the pandemic may increase air pollution risk perception, which in turn amplifies psychological burdens.

##### **Hypothesis 5a**

*The COVID-19 pandemic alleviated mental stress by reducing public perceptions of air pollution risks.*

##### **Hypothesis 5b**

*The COVID-19 pandemic exacerbated mental stress by increasing public perceptions of air pollution risks.*

#### Model III: Self-reported physical symptoms, risk perception, and stress

Finally, we consider the interaction between self-reported physical symptoms, air pollution risk perception, and mental stress. Prior studies indicate that health symptoms related to air pollution—such as respiratory discomfort—heighten both environmental risk perception and psychological strain^48,49^. Integrating these insights, we develop a mediation model in which physical symptoms influence stress both directly and indirectly through risk perception. Here, we focus on the moderating role of the COVID-19 crisis in this pathway.

From a stress appraisal perspective, crises amplify the salience of health-related experiences: identical symptoms may be interpreted as more threatening during an ongoing pandemic than in ordinary times^50^. Similarly, risk amplification theory suggests that contextual cues, such as widespread crisis narratives, can heighten the perceived severity of both symptoms and environmental hazards^51^. Taken together, these perspectives imply that the pandemic may intensify the linkages between physical symptoms, air pollution risk perception, and mental stress.

##### **Hypothesis 6**

*The COVID-19 pandemic strengthened the positive effect of physical symptoms on mental stress, such that individuals with similar symptoms experienced higher stress levels during the crisis compared to before the crisis.*

##### **Hypothesis 7**

*The COVID-19 pandemic strengthened the positive effect of physical symptoms on air pollution risk perception.*

##### **Hypothesis 8**

*The COVID-19 pandemic strengthened the positive effect of air pollution risk perception on mental stress.*

## Results

### Changes in perceived air pollution and mental health before and during the COVID-19 crisis

**Fig. 2:**
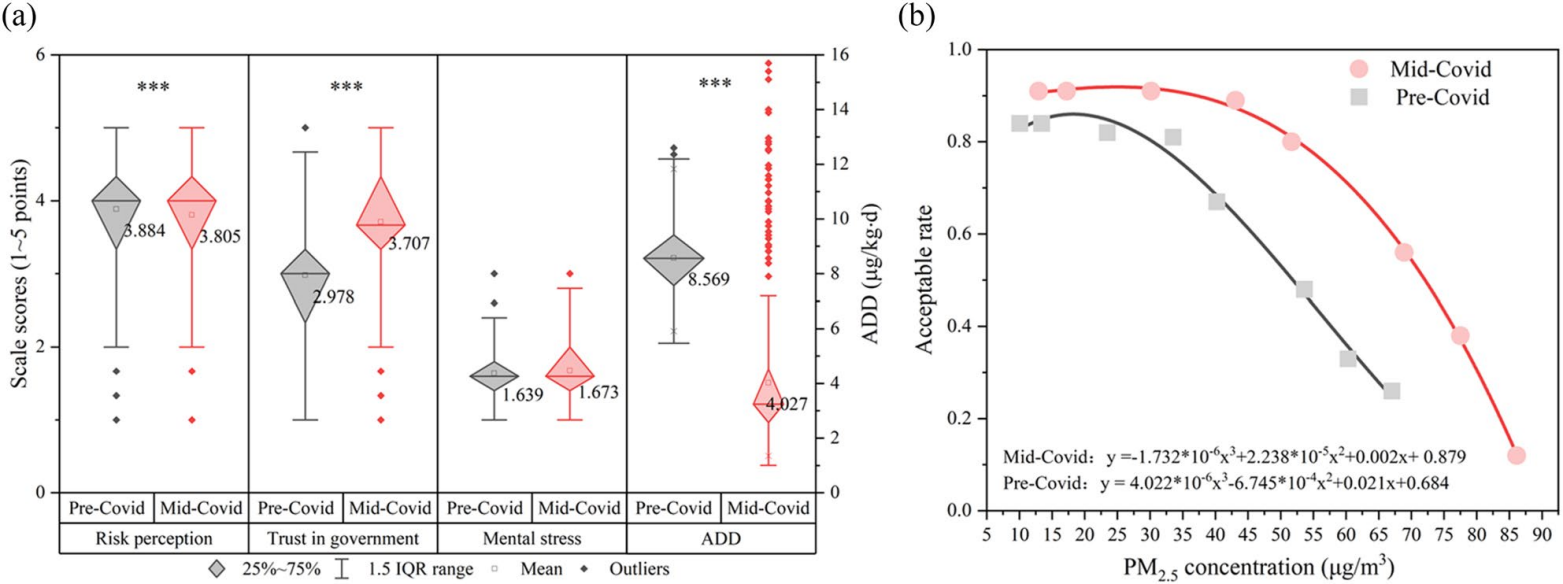
Changes in variables before and during COVID-19.

As shown in Fig. 2a, public risk perception of air pollution was significantly lower in the mid-COVID survey than in the pre-COVID survey (*p* < 0.001), which supports H1a and is inconsistent with H1b. ADD also decreased markedly (*p* < 0.001), whereas trust in government’s capacity to control air pollution increased (*p* < 0.001), consistent with the first parts of H2 and H3. Mean mental stress increased slightly, but this change was not statistically significant (*p* > 0.05). Public familiarity with air pollution rose significantly over time (Table S1), while reports of self-attributed air-pollution-related symptoms remained stable. These results suggest that the pandemic period was characterized by lower perceived air-pollution risk and higher capacity-based trust despite greater familiarity with the issue.

To examine how acceptance varied with pollution levels, we calculated acceptance rates at different PM_2.5_ concentrations and regressed them on concentration to obtain fitted curves (Fig. 2b). In both survey waves, acceptance of PM_2.5_ pollution declined monotonically as PM_2.5_ concentration increased. Across the entire concentration range, however, acceptance was consistently higher in the mid-COVID survey than in the pre-COVID survey. Because both COVID-19 and PM_2.5_ constitute daily risks, this upward shift in acceptance may reflect a re-prioritization of risks during the pandemic, whereby attention and concern were redirected from chronic air pollution to the more salient threat of COVID-19.

### Impact of the COVID-19 crisis on risk perception of air pollution

**Fig. 3:**
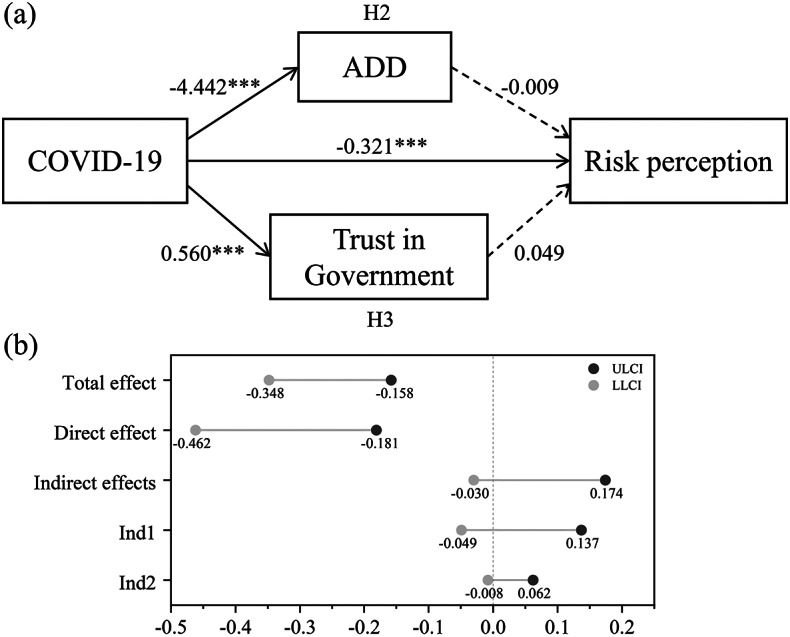
COVID-19 crisis shifted risk perception of air pollution (hypotheses 1–3). (**a**) pathway of Model Ⅰ. (**b**) confidence interval of Model Ⅰ. *p < 0.05, **p < 0.01, ***p < 0.001. ULCI stands for “Upper Limit of the Confidence Interval”, and LLCI stands for “Lower Limit of the Confidence Interval”. When the confidence interval does not contain zero, the result is considered significant. Ind1 and Ind2 respectively represent paths mediated by ADD and capacity-based trust in government.

Regression results (Table S2) and PROCESS mediation models (Fig. 3) consistently showed that the COVID-19 period was associated with a significant decrease in perceived air pollution risk, supporting H1a and not H1b. COVID-19 significantly reduced average daily exposure (ADD) and increased trust in government’s capacity to control air pollution; however, neither ADD nor trust significantly predicted risk perception, and their indirect effects were non-significant in the mediation models (Fig. 3a). The total and direct effects of COVID-19 on risk perception remained significant (Fig. 3b), indicating that the pandemic’s impact on perceived air pollution risk was primarily direct rather than mediated by changes in average daily exposure or capacity-based trust, providing no support for H2 and H3.

**Fig. 4:**
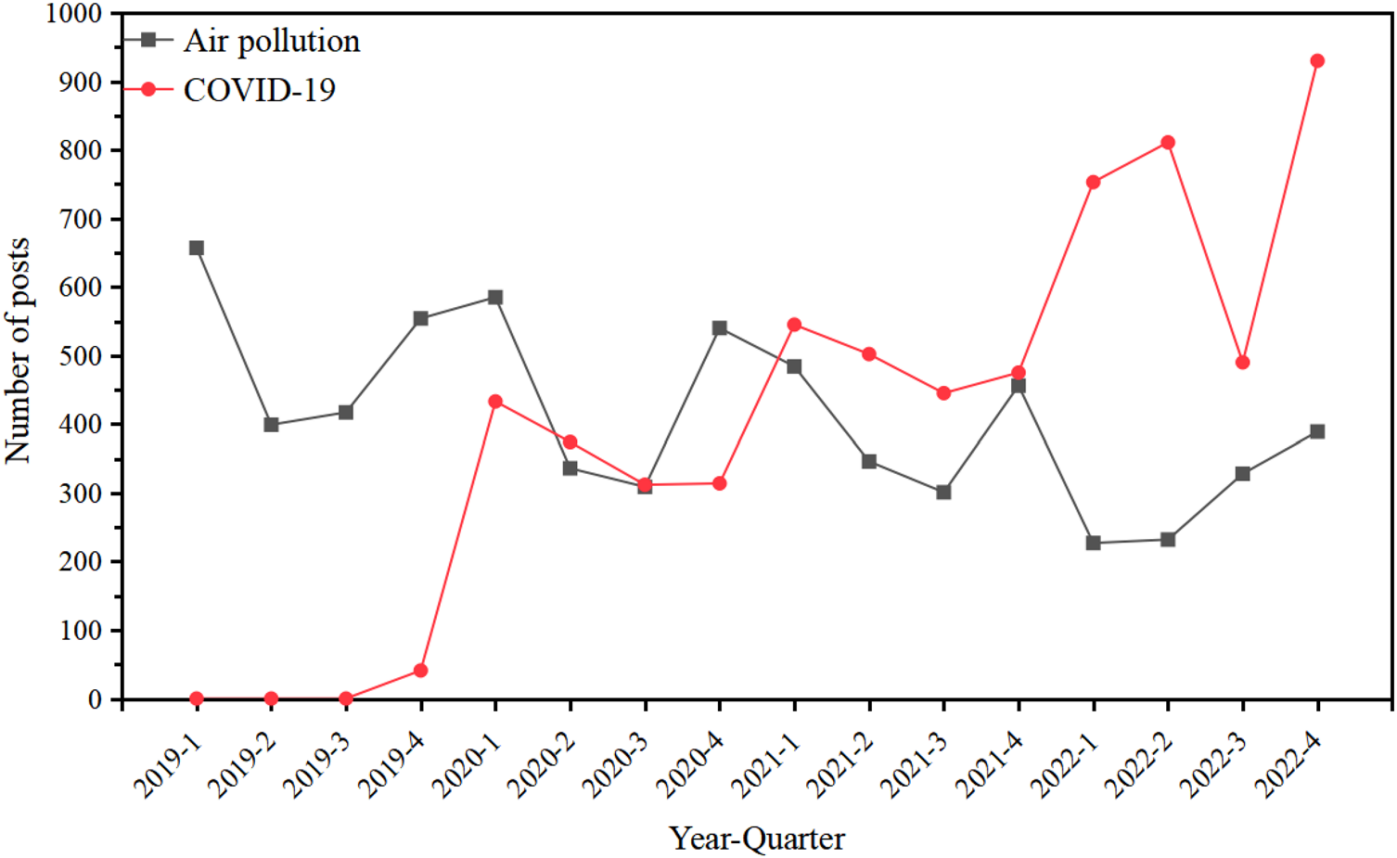
Quarterly Variation in Sina Weibo Posts on “Air pollution” and “COVID-19” in Nanjing from 2019 to 2022.

To further examine how public attention to different risk domains evolved over time, we analyzed quarterly Sina Weibo posts geotagged in Nanjing from 2019 to 2022. The dataset reveals a clear divergence in the temporal trajectories of attention to air pollution and COVID-19. From 2019 through late 2019, air-pollution-related posts dominated the discourse, averaging between approximately 400 and 700 posts per quarter, while COVID-19-related posts were nearly absent. However, beginning in the first quarter of 2020, coinciding with the emergence of COVID-19, posts containing COVID-19 keywords surged sharply to more than 400 per quarter, surpassing air-pollution-related posts. Throughout 2020 and 2021, COVID-19-related attention remained relatively high, fluctuating between roughly 300 and 600 posts per quarter, whereas attention to air pollution showed a gradual decline over the same period. In 2022, COVID-19 attention rose again, peaking at nearly 1000 posts in the fourth quarter, while air-pollution-related posts remained comparatively stable at moderate levels (Fig. 4). This temporal pattern suggests that the emergence and recurrent waves of COVID-19 substantially reshaped the distribution of public attention on social media, at times displacing attention to environmental health concerns such as air pollution. Therefore, the Sina Weibo data from Nanjing provide stronger support for the idea of a finite pool of attention.

Beyond the city-level evidence from Nanjing, World Risk Poll data provide macro-level corroboration that risk salience shifted during the pandemic. Globally, the share of respondents identifying health risks as the greatest threat in daily life increased from 14.67% in 2019 to 19.57% in 2021, whereas the share identifying pollution declined from 0.62 to 0.38% (Table S3). Concern about broad environmental risks also decreased slightly (3.44% to 3.13%). Although patterns varied somewhat across country income groups, the overall direction is consistent with a pandemic-era reallocation of public concern away from pollution-related risks.

### Further impacts on mental health

**Fig. 5:**
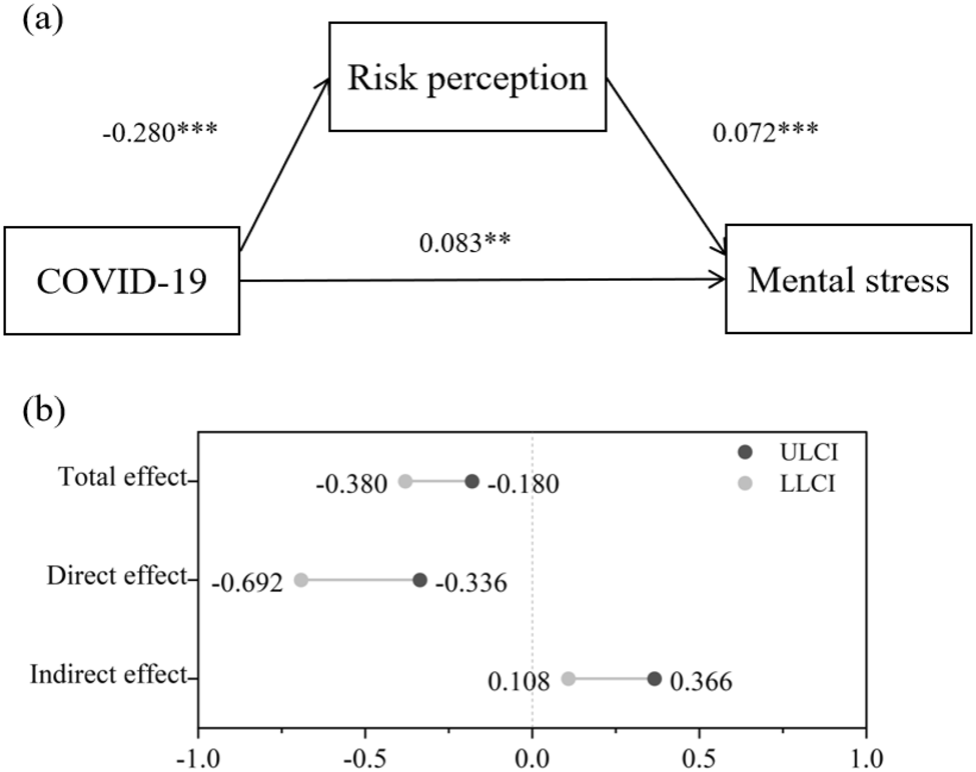
COVID-19 crisis offset part of the stress from air pollution risk perception but overall increased mental stress (hypotheses 4–5). (**a**) pathway of Model Ⅱ. (**b**) confidence interval of Model Ⅱ. See caption of Fig. 3.

Regression results (Table S4) and PROCESS mediation models (Fig. 5) consistently showed that COVID-19 significantly increased mental stress, supporting H4. Figure 5a indicated a significant negative indirect effect via perceived air pollution risk, and the mediation models confirmed that COVID-19 reduced perceived air-pollution risk, which in turn positively predicted mental stress, yielding a significant negative mediation pathway (supporting H5a but not H5b). As shown in Fig. 5b, however, the direct effect of COVID-19 on mental stress remained significantly positive and the total effect was positive, indicating that although changes in air-pollution risk perception offset part of the stress associated with air pollution, the net impact of COVID-19 was still to increase mental stress.

As shown in Fig. 5, COVID-19 indirectly reduced mental stress by lowering perceived risks of air pollution. To test whether this reduction was driven by lower ADD during the pandemic, we examined the mediation chain “ADD—Risk perception—Mental stress.” Results in Fig. S1 show a non-significant total effect, consistent with the rejection of H2, suggesting that objective exposure alone does not drive subjective risk perception.

Therefore, we explored whether self-reported physical symptoms (subjective exposure experience) could serve as a better proxy for objective exposure. As shown in Fig. S2, the total, direct, and indirect effects were all significant, with both the direct and mediated paths from physical symptoms to mental stress being significantly positive. This suggests that subjective discomfort from air pollution, rather than ADD, is associated with risk perception and mental stress, indicating a gap between objective exposure and subjective perception of health impacts.

**Fig. 6:**
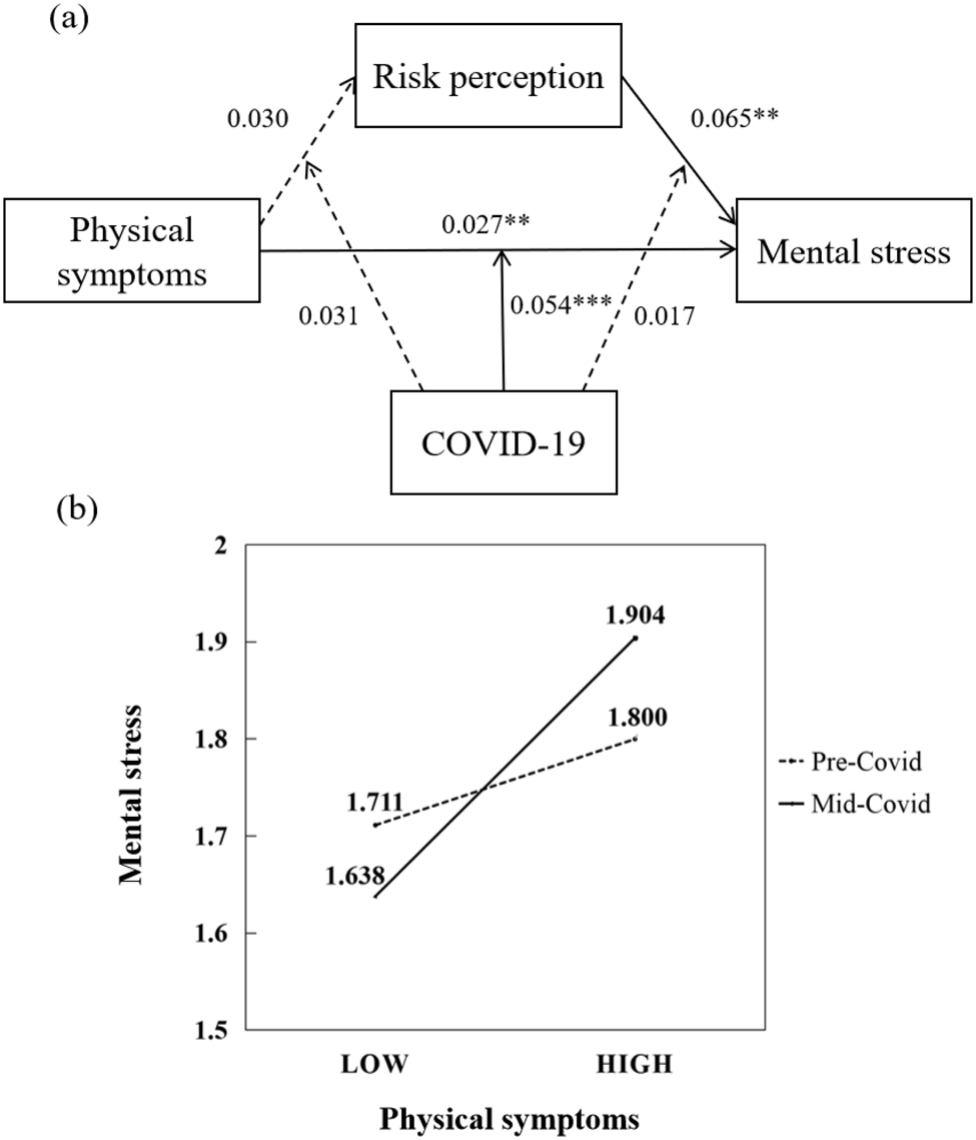
COVID-19 crisis amplified the adverse impact of health threats on mental health (hypotheses 6–8). (**a**) pathway of Model Ⅲ, (**b**) oderating effect of COVID-19. *p < 0.05, **p < 0.01, ***p < 0.001.

We further investigated the role of COVID-19 in this pathway. As shown in Fig. 6, COVID-19 significantly strengthened the relationship between physical symptoms and mental stress, indicating that identical symptoms induced greater psychological burden during the pandemic. This supports H6, while H7 and H8 were not supported. The non-significant results for H7 and H8 indicate that COVID-19 did not amplify the effects of self-attributed air-pollution-related physical symptoms on air-pollution risk perception, nor the effect of air-pollution risk perception on mental stress. Together, these results indicate that crisis contexts may intensify the mental health burden associated with health threats, even if they do not significantly alter the perception pathway itself.

Given that lay respondents may have difficulty clearly distinguishing whether bodily symptoms arise from air pollution or from other sources, we conducted additional sensitivity analyses by decomposing symptoms into those with higher overlap with typical COVID-19 manifestations (COVID-overlapping symptoms, such as cough and chest tightness) and those with lower overlap and more typical pollution-related irritation or allergic features (pollution-typical irritation symptoms, such as allergies and skin itching). We found that, in both groups, the total effects of physical symptoms on mental stress remained significant (Fig. S3), and the strengthening effects of COVID-19 were also significant (Fig. S4). Based on these results, we infer that self-attributed air-pollution-related physical symptoms were significantly and positively associated with mental stress, and that COVID-19 strengthened this association. Furthermore, because more general systemic symptoms also showed significant effects on mental stress, and COVID-19 similarly strengthened this pathway, the amplifying effect of COVID-19 on health anxiety may extend to broader forms of generalized health anxiety, highlighting the heightened vulnerability of public mental health under crisis conditions. The specific direct effect values for the three categories of physical symptoms before and during COVID-19 are reported in Table S5.

## Discussion

This study, drawing on two comparable surveys conducted pre-Covid and mid-Covid, systematically examined how the COVID-19 crisis reshaped public perceptions of air pollution risks and mental stress. Drawing on two repeated cross-sectional surveys conducted before COVID-19 (2018–2019) and during the COVID-era (2022), the analysis highlights the complex role of acute crises: they can shift concern away from chronic risks, thereby reducing risk perception, while simultaneously amplifying the psychological burden of existing health problems, offering new insights into cross-crisis spillover and societal adaptation to compound risks. Importantly, we interpret the between-wave differences as evidence of COVID-era associated shifts rather than strict individual-level causal effects, given the repeated cross-sectional design and the change in survey mode (face-to-face vs. online). To strengthen comparability, we complemented the main analyses with measurement invariance tests (Table S6) and sensitivity checks using raking calibration weights (Table S7), which together reduce concerns about scale non-equivalence and mode-related compositional differences.

Findings show that the COVID-19 pandemic significantly reduced public perception of health risks related to air pollution (Figs. 2a, 3, Table S3), supporting Hypothesis 1a. Therefore, our results are consistent with the direction predicted by the finite-pool-of-worry hypothesis^2,17,52^. While this does not completely refute the affect-generalization theory, the latter appears to exert a weaker effect. The two mechanisms may coexist^11^, but the decline effect dominates the overall outcome. As an acute and salient survival threat, COVID-19 consumed public cognitive and emotional resources, reducing concern to long-term environmental risks such as air pollution^37^. Our Nanjing social-media evidence should be interpreted as complementary process evidence rather than a direct measure of individual-level worry. Specifically, the Weibo time series shows a clear temporal divergence in issue attention: air-pollution-related posts dominated in 2019, whereas COVID-19-related posts surged from early 2020 and remained elevated through 2021, with another peak in late 2022 (Fig. 4). This pattern indicates that the information environment and the public salience of air pollution changed substantially during the pandemic, thereby providing process evidence that complements the survey-based decline in perceived air-pollution risk. To enhance reproducibility, we conducted a manual validation by independently coding a random subset of posts; the two coders achieved high agreement, supporting the reliability of the content filtering and labeling procedure (Table S8). On the other hand, this may also reflect a risk comparison effect^53,54^: when juxtaposed with a life-threatening infectious disease, the health risks posed by air pollution were relatively “downplayed,” as reflected in the increased public acceptance documented in Fig. 2b. However, processes of risk trade-off are more commonly observed in risk decision-making contexts^33^. When two risks are perceived as belonging to a common decision space—such as nuclear power and climate change, where nuclear power may be viewed as a means of mitigating climate change—public judgments are more likely to involve explicit trade-offs or conditional prioritization^27,55^. By contrast, when risks are not intuitively framed as direct substitutes, such as COVID-19 and air pollution, a decline in concern is more plausibly interpreted as attentional or affective crowding-out rather than deliberate reprioritization^18,56^.

World Risk Poll data suggest that the COVID-19 outbreak crowded out public concern about environmental risks such as air pollution, with a clear income-gradient pattern. Importantly, because the World Risk Poll asks respondents to identify the single greatest source of risk in their daily lives, these cross-income differences are better understood as differences in which risks rose to the top of public concern in daily life rather than as simple differences in environmental concern. In low-income countries, the sharpest decline in environmental risk salience alongside no corresponding increase in health risk salience is more consistent with a risk hierarchy or livelihood-first pattern: under conditions of greater material insecurity, limited cognitive and emotional resources may be directed first toward income, food, safety, and basic survival concerns, thereby pushing both environmental and health risks downward in the overall ranking of daily-life threats^57^. In upper-middle-income countries, where pollution had remained a visible and familiar everyday issue, the pandemic appears to have generated a clearer substitution effect, redirecting attention from environmental hazards toward the acute health emergency^2^. In high-income countries, by contrast, environmental concern may be more institutionally embedded; thus, the decline in narrow pollution concern alongside relatively stable broad environmental concern is more consistent with issue evolution than with simple crowding-out, suggesting a shift from specific pollution risks toward broader environmental or climate-related frames^58^. These interpretations remain conceptual rather than causal, but they help explain why the same pandemic shock produced different salience patterns across national income contexts. Crucially, these global patterns are not driven solely by differences in country coverage across waves: sensitivity analyses restricting the World Risk Poll to countries appearing in both 2019 and 2021 produced substantively consistent results (Table S9).

Although pandemic control measures substantially reduced objective PM_2.5_ exposure levels, the mediating effect of ADD was not supported (H2 rejected; Fig. 3). This may be because the public’s attention was primarily directed toward the acute and urgent threat of the virus, leaving the objective change in ADD largely unnoticed. Further analyses indicate that objective exposure (ADD) itself is not a key driver of risk perception of air pollution or its psychological consequences (Fig. S1). Instead, the pathway “physical symptoms—risk perception—mental stress” is strongly supported (Fig. S2), highlighting that public risk perception depends heavily on subjective experiences and contextual interpretations^36^. When individuals personally experience discomfort, their risk perception is more likely to increase, thereby intensifying mental stress. These findings point to an “exposure-perception paradox”^59^: risk perception is not a mirror reflection of objective exposure, but a psychologically and socially constructed judgment shaped by embodied experience, contextual cues, and affective signals. First, the stronger role of self-reported symptoms is consistent with the availability heuristic. Symptoms are vivid, immediate, and emotionally salient, whereas estimated exposure metrics such as ADD—derived from time-activity reports and parameter assumptions—are less perceptible to laypeople and therefore less likely to enter intuitive risk judgments or trigger stress responses. Second, this pattern also aligns with constructionist perspectives on risk, which emphasize that perceived risk is actively shaped through interpretation, communication, and social meaning rather than being read directly from environmental conditions^60^. In this sense, the gap between objective exposure and perceived risk reflects not measurement failure alone, but the fact that subjective appraisal is more strongly influenced by psychosocial processes such as media attention, public discourse and cognitive bias^51,61^. Psychological distance may further widen the gap between objective exposure and subjective appraisal, because the health implications of exposure are often probabilistic, delayed, and difficult to attribute^62^. As a result, such risks may feel less immediate, and thus less psychologically stressful, than directly experienced symptoms. By contrast, bodily discomfort reduces this distance by turning an abstract environmental hazard into a personally felt experience. Taken together, these findings suggest that the “exposure effect” in air-pollution risk perception is conditional rather than automatic: objective conditions matter mainly when they are translated into salient experiences or socially meaningful signals, which in turn are more likely to be appraised as personally threatening and therefore to intensify mental stress.

This study shows that the COVID-19 crisis increased public trust in the government’s capacity to control air pollution, suggesting that capacity-based trust accumulated during an acute emergency can spill over across policy domains. Effective governance during emergencies often serves as a strong signal of institutional competence^63,64^. Our results suggest that public recognition of the government’s crisis management capacity generalized to the environmental domain, likely through an institutional “halo effect” that bolstered confidence in air pollution governance. This mechanism resonates with the concept of “trust spillover,” whereby credibility gained in one policy domain spills over into others^20^. However, this increase in trust did not translate into a significant reduction in perceived air-pollution risk (H3 rejected). Supplementary regression analysis further showed that trust in government’s capacity to control air pollution was not a significant predictor of air-pollution risk perception (Table S10). This pattern suggests that trust spillover and risk-appraisal spillover should be distinguished: capacity-based trust may generalize across domains at the level of institutional evaluation, without necessarily altering cognitive appraisals of risk. Whether trust spillover extends into risk-appraisal spillover may depend both on the specific dimension of trust involved and on the type of hazard being evaluated^65^.

One possible explanation concerns the dimension of trust measured in this study. Institutional trust is often conceptualized as a multidimensional construct, including perceived capacity, integrity, and benevolence^66^. In risk regulation and environmental risk communication, public trust and credibility have also been linked not only to knowledge and expertise, but also to openness, honesty, fairness, concern, and care^67^. Capacity-based trust may signal that the government is capable of managing air pollution, but it may not be sufficient to alter individuals’ cognitive appraisal of the risk itself. By contrast, integrity-based trust may influence whether people believe official risk information and disclosure^40^, while benevolence-based trust may be more closely related to feelings of being protected, reassured, and psychologically secure^68^. Because these dimensions were not directly measured in this study, this interpretation should be regarded as a theoretical possibility rather than an empirical conclusion. A second explanation concerns the nature of air pollution as a chronic and everyday environmental hazard. Unlike more abstract and less directly perceptible hazards, air pollution is often appraised through sensory cues, bodily discomfort, media exposure, and local environmental experience^69^. As a result, for chronic and routine environmental hazards, risk judgments may remain more strongly anchored in lived experience than in capacity-based trust. In this context, capacity-based trust may function more as a heuristic for policy support and compliance than as a determinant of cognitive risk assessment^43^. By contrast, for more abstract, unfamiliar, or technically complex hazards such as nuclear power, public risk judgments may depend more heavily on broader institutional trust as a heuristic. Under such conditions, crisis-induced trust spillover may be more likely to extend into risk-appraisal spillover and thereby shape public attitudes^21^. Taken together, our findings indicate an important boundary condition for cross-domain trust effects: crisis management can strengthen confidence in government capacity, but this does not necessarily reduce perceived risk for chronic, everyday environmental hazards.

The study reveals a dual effect of public health crises on stress: On the one hand, the pandemic reduced perceptions of air pollution risks, which indirectly eased mental stress (H5a supported). This reflects an extension of the COVID-19-induced reduction in air-pollution risk perception into the mental health domain (Fig. 5). On the other hand, the pandemic itself markedly heightened stress levels (H4 supported), consistent with the well-documented psychological toll of major public emergencies^44^. The coexistence of these opposing effects illustrates a key distinction: risk perception is domain-specific, while stress is shaped by multiple sources. Whereas our study zooms in on air pollution as a single hazard, stress represents a broader response to threats across everyday life—ranging from health and finances to work and social relations^47,70^. In this sense, COVID-19 functioned less as an indirect driver of stress through altered environmental risk perceptions than as a direct, overarching stressor linked to health threats, social isolation, and economic uncertainty^9,71^. This also signals the need for future research that employs more comprehensive stress measures and models spanning multiple life domains. Methodologically, our primary inference here relies on theory-driven hierarchical (blockwise) regression and PROCESS-based mediation/moderation with bootstrapping; stepwise selection results are provided only as a robustness check in the Supplement to avoid over-reliance on data-driven variable selection.

Importantly, the crisis acted as a contextual amplifier. While it diverted public concern away from pollution risks (H1a), it simultaneously raised public sensitivity to personal health conditions. COVID-19 significantly strengthened the relationship between physical symptoms and mental stress, indicating that similar symptom experiences imposed a greater psychological burden during the pandemic. Sensitivity analyses further showed that, even when symptoms were decomposed into those with higher overlap with typical COVID-19 manifestations and those more typical of pollution-related irritation or allergy, both the symptoms—stress association and the strengthening effect of COVID-19 remained significant. These findings suggest that the amplified symptoms-stress relationship cannot be fully explained by air-pollution attribution alone, and may partly reflect broader health-related anxiety under crisis conditions. This helps explain the seemingly paradoxical pattern in Fig. 5: reduced concern about pollution produced a stress-buffering effect, while heightened sensitivity to symptoms produced a stress-amplifying effect, with the two partially offsetting each other. Overall, these findings show that crises not only redistribute cognitive and emotional resources across risks but also heighten overall vulnerability to health-related stressors. Notably, the questionnaire did not ask about generic symptoms in isolation; rather, respondents were explicitly asked to report the “physiological reactions to air pollution” they experienced based on personal experience. Thus, the symptom measure captures self-attributed bodily discomfort within an air-pollution frame rather than clinically verified etiology. In real-world compound-crisis settings, lay respondents may not fully disentangle symptom sources, and this ambiguity is itself part of the context in which risk perception and mental stress are formed. Under overlapping COVID-19 and air-pollution conditions, such symptom experiences may also have been colored by broader health anxiety, and should therefore be interpreted as perception-relevant subjective experiences rather than strict pollution-specific indicators.

This study has several limitations. First, symptom attribution may have remained ambiguous during the pandemic. Although the questionnaire explicitly asked respondents to report physiological reactions they associated with air pollution, some bodily discomfort may also have been interpreted in relation to COVID-19 or broader health anxiety. Future studies should incorporate more fine-grained attribution measures, such as open-ended attribution questions, or competing-cause ratings to better distinguish symptom sources. Second, although both survey waves were broadly representative of the Nanjing population, they did not follow the same individuals and were administered in different modes (face-to-face before COVID-19 versus online during COVID-19). These features limit strict causal inference and may introduce residual compositional or mode-related differences, despite our measurement invariance tests and sensitivity analyses with post-stratification weights. In particular, because the mediation analyses rely on repeated cross-sectional rather than longitudinal data, the indirect pathways should be interpreted as theory-informed associations rather than definitive causal mechanisms. Future research should adopt longitudinal panel designs with consistent survey modes to strengthen causal inference. Third, the auxiliary data used for triangulation also have limitations. Our Weibo analysis relies on keyword dictionaries and AI-assisted filtering, and may still be influenced by platform dynamics and posting behaviors. Likewise, although the World Risk Poll provides valuable global corroboration, cross-wave differences in participating countries and response categories may affect comparability, even though we constructed harmonized measures and repeated analyses in overlapping country samples. Finally, although our data are consistent with both risk trade-off and the finite pool of worry account, our survey did not directly measure priority ranking. We therefore cannot strictly determine which mechanism is statistically dominant in explaining the decline in perceived air-pollution risk. Future research could incorporate explicit measures of risk prioritization, such as priority-ranking items, trade-off questions, or choice-based experiments, to determine which mechanism dominates in the case of COVID-19 and air pollution.

The aim of communication under compound-crisis conditions is not only to preserve the public’s capacity to protect themselves against multiple risks, but also to safeguard mental well-being. This study therefore has several practical implications for compound-crisis governance. First, because acute crises may crowd out attention to chronic environmental hazards, policymakers and media organizations should continue to maintain awareness of background environmental risks even during emergencies. Compound-risk communication should avoid zero-sum narratives and instead adopt dual-track messaging: one channel for the acute emergency and another for stable, lower-frequency reminders about ongoing environmental risks, especially for vulnerable groups. Second, the exposure-perception paradox highlights risks in both directions. When actual exposure is underestimated, protective responses may be insufficient; when perceived risk exceeds actual exposure, individuals may misattribute other bodily symptoms to air pollution, thereby increasing psychological burden, particularly under compound-crisis conditions. Communication should therefore link monitoring data with symptom-relevant guidance rather than reporting PM_2.5_ or AQI values alone, including tailored advice for sensitive groups, symptom self-check guidance, and recommendations on when protective action or medical attention is warranted. Third, because compound-crisis conditions can amplify the psychological burden associated with physical symptoms and complicate symptom interpretation across coexisting risks, mental-health responses should be layered and integrated with multi-risk communication. For the general population, low-intensity stress-management tools and clear, non-alarmist communication may help reduce uncertainty under overlapping threats. For individuals reporting physical discomfort or persistent distress, brief screening, active follow-up, and stepped referral should be combined with calibrated explanations of symptoms in the context of coexisting acute and chronic risks^72^. Overall, effective compound-crisis governance requires an integrated communication system that both sustains awareness of long-term environmental risks and addresses the mental-health consequences of acute emergencies.

By integrating survey evidence, social media attention dynamics, and global risk-salience data, this study offers a multi-scale account of how crises reallocate public concern and reshape the perception-stress pathway. The findings show that acute emergencies can reduce perceived chronic environmental risks while simultaneously increasing overall psychological burden, and that subjective symptoms matter more than objective exposure changes in shaping risk perception and mental stress. Although effective crisis management increased capacity-based trust in government, this trust did not significantly reduce perceived air-pollution risk. Together, these findings highlight the need for compound-crisis governance that integrates sustained risk communication, targeted trust building, and mental health support.

## Methods

### Questionnaire surveys

#### Research design and sample

Between January 2018 and July 2019, we conducted a face-to-face survey in Nanjing and obtained 508 valid responses from adult participants^69^. These pre-Covid data were incorporated into the present study for comparison with a follow-up survey conducted during COVID-19. Due to pandemic-related restrictions, we commissioned “Netease Dingwei” to administer an online survey to adults in Nanjing using a random sampling approach between June and December 2022, yielding 500 valid responses. Informed consent was obtained from all participants prior to participation, and respondents were informed that participation was voluntary and that they could discontinue at any time without penalty. In both waves, respondents were randomly drawn from all 11 administrative districts. We compared the sample sizes from each district with district-level population sizes to ensure representativeness (Table S11). Although the two surveys did not track the same individuals, the samples provide comparable and representative coverage of Nanjing residents. Descriptive statistics of sociodemographic characteristics are reported in Tables S12 and S13. All methods were carried out in accordance with relevant guidelines and regulations and complied with the Declaration of Helsinki. The study protocol was reviewed and approved by the Social Science Subcommittee of the Science and Technology Ethics Committee, Nanjing University (approval no. NJUSOC202512005).

We conducted sensitivity analyses to address concerns that the two waves, as independent cross-sectional samples, may differ in sample composition. In addition to adjusting for key sociodemographic characteristics in all models, we re-estimated the main risk-perception specification using raking (iterative proportional fitting) weights that calibrated the sample marginals of gender, age group, and education to Nanjing’s citywide population benchmarks. The estimated COVID-19 effect on risk perception remained negative and statistically significant after reweighting (Table S7). A detailed description of the weighting procedure is presented in Text S1.

#### Questionnaire and variable measurements

The questionnaire was adapted from the risk perception psychometric scale developed by Huang et al., with minor revisions for this study^73,74^. To ensure comparability across waves, the measurement scales were kept consistent across both the pre-Covid and mid-Covid surveys^69^. The instrument comprised four sections (Text S2). The COVID-19 period was represented by a binary indicator denoting whether the respondent was surveyed during the COVID-19 era (1 = 2022 wave; 0 = 2018–2019 wave).

##### Section 1: Risk perception and mental stress

The first section included a 12-item risk perception scale and an 8-item mental stress scale^75^, both measured on 5-point Likert scales. We conducted exploratory factor analysis on the 12 risk perception items using principal component extraction and varimax rotation. Four factors were identified: familiarity of air pollution, perceived health risks, perceived controllability, and capacity-based trust in government. One item (Q7) was removed due to cross-loadings > 0.4 (Table S14). The KMO and Bartlett’s test supported sampling adequacy (Table S15). Cronbach’s alpha indicated good reliability for all factors except perceived controllability (Cronbach’s alpha < 0.6), which was excluded from subsequent analyses (Table S16. Confirmatory factor analysis supported the structural validity of the remaining three dimensions (Table S17). Measurement invariance was tested across the pre- and mid-COVID survey waves using multi-group CFA with ordered indicators (WLSMV, theta parameterization). The results supported configural, metric (equal loadings), and scalar invariance (equal loadings and thresholds) for the air-pollution risk perception scale (Table S6), indicating that the construct was measured comparably across waves.

*Risk perception of air pollution.* This construct captures respondents’ perceived health risk associated with air pollution. It was measured using three items: (1) “Do you consider smog/haze an ordinary risk or a frightening risk?” (2) “If smog/haze weather occurs locally, how severe do you think its impact on your personal health would be?” and (3) “Do you believe the health risks from severe smog/haze weather could be fatal?”.

*Trust in government’s capacity to control air pollution.* This construct primarily reflects capacity-based trust in government, rather than a broader multidimensional form of institutional trust. It was measured by three items: (1) “Do you consider the national air quality standards to be strict?” (2) “Do you believe the government takes corresponding emergency measures during severe smog/haze weather?” and (3) “Do you trust the government to effectively manage smog/haze weather?”.

For the mental stress scale, reliability analyses suggested that three items (Q5: “Do you dislike going out alone?”; Q7: “Do you feel more relaxed indoors?”; Q8: “Do you feel particularly worried when a family member returns home late?”) contributed to low internal consistency. After removing these items, the remaining 5-item scale achieved Cronbach’s alpha values above 0.60 in both waves. Accordingly, this reduced 5-item version was used as the outcome measure for mental stress (Table S18).

##### Section 2: Maximum acceptable risk level (MARL)

The second section applied the MARL framework to assess respondents’ tolerance for reductions in PM_2.5_ concentrations over the next three years^73,76^. Respondents rated the acceptability of projected PM_2.5_ concentrations under policy scenarios (2017 and 2022) on a 5-point scale from “fully acceptable” to “unacceptable.” Concentrations were derived from the 2012 baseline (67 µg/m^3^) with reduction rates ranging from 85 to 10%. Detailed calculation procedures are provided in the Text S3.

##### Section 3: Average daily exposure (ADD)

The third section recorded respondents’ typical daily indoor and outdoor exposure durations during the pandemic to estimate average daily PM_2.5_ exposure (ADD). ADD was Calculated using the U.S. EPA-recommended formula^77^, adjusted for indoor/outdoor exposure time, PM_2.5_ concentration, inhalation rate (IR), and body weight (BW). The equation is:

$$ADD=({C}_{1}\times {IR}_{1}\times {EF}_{1}/24+{C}_{2}\times R\times {IR}_{2}\times {EF}_{2}/24)/BW$$

where C_1_ and C_2_ are the average outdoor and indoor PM_2.5_ concentrations (µg/m^3^; data from Qingyue Open Data, https://data.epmap.org). IR_1_ and IR_2_ are inhalation rates during outdoor (15.8 m^3^/d for males, 14.1 m^3^/d for females) and indoor activities (15.1 m^3^/d for males, 13.5 m^3^/d for females)^78^; EF_1_ and EF_2_ are the durations of outdoor and indoor exposure (h; questionnaire data); R is the indoor/outdoor concentration ratio (0.85)^79^; and BW is body weight (kg; questionnaire data).

##### Section 4: Covariates and physical symptoms

The fourth section collected sociodemographic information, including gender, age, education, and monthly income, and further captured residential environment and self-reported physical symptoms. Physical symptoms included cough, chest tightness/difficulty breathing, headache/dizziness, throat discomfort (e.g., soreness or hoarseness), allergies, lethargy, skin itching, dry or watery eyes, nasal congestion/runny nose, as well as two psychological symptoms (anxiety due to worry and irritability). In the main analysis, we excluded the two psychological symptoms and used the remaining nine items as a stricter indicator of physical symptoms (Figs. S2; 6). The physical symptom score was calculated as the number of symptoms, with higher scores indicating greater symptom burden. For sensitivity analysis, we constructed two alternative symptom indicators. The first was a high-overlap symptom index (COVID-overlapping symptoms), consisting of symptoms that could be more easily confounded with COVID-19-related manifestations, including cough, chest tightness/difficulty breathing, headache/dizziness, throat discomfort, lethargy, and nasal congestion/runny nose. The second was a low-overlap symptom index (pollution-typical symptoms), consisting of symptoms more characteristic of pollution-related irritation or allergic responses, including allergies, skin itching, and dry or watery eyes. The number of symptoms in each group was used as an alternative indicator in sensitivity analyses (Figs. S3, S4). Control variables included physical symptoms, familiarity of air pollution, and key sociodemographic factors (age, gender, education, income, and residential environment), which were entered into all regression and mediation models.

#### Data analysis

We first conducted descriptive statistics and between-wave comparisons using independent-samples t-tests and Mann–Whitney U tests. When Levene’s test indicated unequal variances, we used Mann–Whitney U tests as an alternative to t-tests.

Second, we tested hypotheses using theory-driven hierarchical multiple linear regression, examining changes in R^2^ as blocks of variables were added sequentially^80^. For transparency, stepwise results are also provided as a sensitivity check in the Tables S2 and S4. Variance inflation factor (VIF) results indicated no multicollinearity in the models (Table S19).

Third, we applied the PROCESS macro to test the three theoretical models and eight hypotheses^81^. Hypotheses 2, 3, and 5 were tested using simple mediation models (PROCESS Model 4), with Hypotheses 1 and 4 corresponding to the direct effects within these models. The moderating role of COVID-19 (Hypotheses 6–8) was further tested using PROCESS Model 59. All mediation and moderation analyses used 5000 bootstrap resamples to compute bias-corrected confidence intervals.

### Social media data

We conducted a social media analysis to examine temporal changes in public attention to COVID-19 and haze-related air pollution in Nanjing, China. Two topic-specific keyword dictionaries were constructed based on terminology commonly used in public health and environmental research. The COVID-19 category included “COVID-19”, “epidemic”, “positive casenucleic acid test” and “coronavirus”. The air pollution category included “haze”, “smog”, “air pollution”, and “air quality”.

Using the Sina Weibo Open API, we retrieved all publicly available posts geotagged in Nanjing between 1 January 2019 and 31 December 2022. To improve data quality, all collected posts were first processed using the DeepSeek-V2 model via API access. Based on publisher names, locations, and post content, this AI-assisted screening step was used to remove clearly irrelevant content, including formal announcements or science-popularization posts from government or professional institutions, finance- and stock-related posts, advertisements and marketing content, celebrity/entertainment gossip, and false keyword matches caused by retrieval errors. To reduce potential platform noise around the early emergence of COVID-19, we excluded anomalous posts containing COVID-19-related keywords prior to November 2019, which likely reflected API instability or timestamp inconsistencies. We then conducted a manual validation step. Two independent coders labeled a random subset of posts to assess the reliability of the coding scheme and to verify the distinction between official/institutional accounts and individual/personal accounts. In this validation process, accounts were treated as official/institutional when the account name indicated a governmental, media, organizational, or other institutional identity, or when the posts mainly took the form of formal announcements, policy notices, official updates, or news-style reports. Posts containing finance-related, advertising, entertainment, or other clearly irrelevant content were also excluded. Inter-coder agreement was high (Table S8), supporting the consistency of the filtering and labeling scheme.

After AI-assisted screening and manual validation, 12,985 posts were retained. We aggregated the cleaned data at a quarterly resolution and calculated, for each quarter, the total number of COVID-19-related and air-pollution-related posts. The resulting quarterly time series provides a consistent basis for comparing public attention to infectious disease risks and air pollution risks in Nanjing over the 2019–2022 period.

### World risk poll

Global-scale data were drawn from the World Risk Poll conducted by the Lloyd’s Register Foundation in collaboration with Gallup^82^. The survey uses multistage stratified probability sampling and is designed to be globally representative. We analysed two cross-sectional waves: 2019 (pre-Covid) and 2021 (mid-Covid). The 2019 wave included 154,195 respondents from 139 countries, and the 2021 wave included 125,911 respondents from 121 countries.

The key outcome was respondents’ choice of “the greatest risk to your safety in daily life.” To capture changes in concern about environmental pollution, we constructed two measures: (1) narrow pollution, defined as the proportion selecting “pollution”; and (2) broad environmental risk, defined as the combined proportion selecting “pollution,” “climate change or severe weather events,” or “non-weather-related disasters,” thereby accounting for minor response-category changes across waves. In addition, to characterise broader shifts in risk salience, we constructed a broad health risk variable by aggregating all explicitly health-related options: in 2019, “personal health/illness,” “drugs, alcohol, smoking,” and “mental health/stress/exhaustion”; in 2021, the “COVID-19” option was added to this set^83^.

For global-scale data from the World Risk Poll, we first conducted descriptive analyses and then performed Chi-square tests stratified by World Bank Income groups (Table S3) to examine heterogeneity in these changes. As a sensitivity check for differences in country coverage between waves, we repeated analyses restricting to countries appearing in both waves and obtained consistent patterns (Table S9).

## Supplementary Information

Supplementary Information.

## Data Availability

All relevant data are within the manuscript and its Supplementary Information files. The data that support the findings of this study are available upon request from the corresponding author, by sending email to Lei Huang; Email: huanglei@nju.edu.cn.
