# Supplementary material for: Cross-crisis effects of COVID-19 on air pollution risk perception and mental stress
Source: Sci Rep. 2026 Jun 2;16:25071. doi: 10.1038/s41598-026-53900-x (PMC13462863; doi:10.1038/s41598-026-53900-x)
Supplement: Supplementary file 1 [file 41598_2026_53900_MOESM1_ESM.pdf]

Supporting Information for

Cross-Crisis Effects of COVID-19 on Air Pollution Risk Perception and Mental Stress

This PDF file includes:

Text S1 to S3
Figures S1 to S4
Tables S1 to S19

Text S1. Post-stratification (Raking) Sensitivity Analysis.

To examine whether differences between the two survey waves—collected as independent cross-sectional samples—could bias the estimated association between COVID-19 and risk perception via shifts in sample composition, we conducted a post-stratification sensitivity analysis using raking (iterative proportional fitting). We constructed calibration weights to align the sample marginal distributions of gender (male/female), age group (six categories: ≤20, 21–29, 30–39, 40–49, 50–60, ≥60), and education (three categories: high school or below, high school, college or above) to Nanjing's citywide population margins from the 2020 census statistics. For education, we harmonized population benchmarks to the three-category scheme by combining the primary- and middle-school groups into "high school or below" and rescaling the remaining categories to proportions.

Raking was implemented separately within each wave (pre-COVID vs. during-COVID), starting from equal base weights (w = 1) and iteratively adjusting weights until convergence on all targeted margins. To improve numerical stability and limit the influence of extreme weights, we normalized the resulting weights within wave to have mean 1 and winsorized weights at the 99th percentile. We then re-estimated the main risk-perception regression model—risk perception regressed on COVID-19, familiarity, trust in government, and covariates (gender, age, education, income, physical symptoms, and living environment)—using the calibrated weights with design-based standard errors (R package survey, rake() and svyglm()). The results were robust: the coefficient of COVID-19 remained negative and statistically significant in the weighted specification (Table S6).

Text S2. Questionnaire on Nanjing Public Perception of Air Pollution.

Part I

1. Do you pay attention to information related to smog/haze weather events? [Single choice]
   ○ Do not pay attention at all ○ Pay little attention ○ Neutral ○ Pay moderate attention ○ Pay full attention
2. Are you aware of the health hazards posed by smog/haze weather? [Single choice]
   ○ Unaware ○ Slightly aware ○ Neutral ○ Moderately aware ○ Very aware
3. Do you believe you can find out about the occurrence of smog/haze weather in a timely manner? [Single choice]
   ○ Completely unable ○ Unlikely ○ Neutral ○ Quite likely ○ Completely able
4. Do you consider smog/haze an ordinary risk or a frightening risk? [Single choice]
   ○ Ordinary ○ Somewhat ordinary ○ Neutral ○ Somewhat frightening ○ Frightening
5. If smog/haze weather occurs locally, how severe do you think its impact on your personal health would be? [Single choice]
   ○ Not severe ○ Slightly severe ○ Neutral ○ Moderately severe ○ Very severe
6. Do you believe the health risks from severe smog/haze weather could be fatal? [Single choice]
   ○ Completely impossible ○ Unlikely ○ Neutral ○ Quite likely ○ Completely possible
7. Do you believe you have the ability to avoid the harm caused by smog/haze weather? [Single choice]
   ○ Completely unable ○ Unlikely ○ Neutral ○ Quite likely ○ Completely able
8. Do you believe methods like reducing pollution source emissions can prevent the occurrence of smog/haze weather? [Single choice]
   ○ Completely cannot ○ Unlikely ○ Neutral ○ Quite likely ○ Completely can
9. Do you believe methods like vehicle restrictions can prevent the occurrence of smog/haze weather? [Single choice]
   ○ Completely cannot ○ Unlikely ○ Neutral ○ Quite likely ○ Completely can
10. Do you consider the national air quality standards to be strict? [Single choice]
   ○ Not strict ○ Somewhat not strict ○ Neutral ○ Somewhat strict ○ Very strict
11. Do you believe the government takes corresponding emergency measures during severe smog/haze weather? [Single choice]
   ○ Not at all ○ Rarely ○ Neutral ○ Moderately ○ Completely
12. Do you trust the government to effectively manage smog/haze weather? [Single choice]
   ○ Completely distrust ○ Somewhat distrust ○ Neutral ○ Somewhat trust ○ Completely trust
13. Have you ever experienced inexplicable fear in enclosed spaces like stores or elevators? [Single choice]
   ○ Never ○ Occasionally ○ Often
14. Are you afraid of heights? [Single choice]
   ○ Not at all ○ A little ○ Very afraid
15. Do you feel panic in crowds? [Single choice]
   ○ Never ○ Occasionally ○ Always
16. Do you worry about contracting difficult-to-cure diseases? [Single choice]
   ○ Never ○ Occasionally ○ Often
17. Are you unwilling to go out alone? [Single choice]
   ○ No ○ Yes
18. When riding on uncrowded buses, subways, or trains, do you feel uncomfortable? [Single choice]
   ○ Not at all ○ A little ○ Very uncomfortable
19. Do you feel more relaxed indoors? [Single choice]
   ○ No noticeable feeling ○ Occasionally ○ Not at all
20. Do you become especially worried when family members return late? [Single choice]
   ○ No ○ Yes

Part II

The following figure shows cities under different PM2.5 concentrations, providing you with a visual reference:

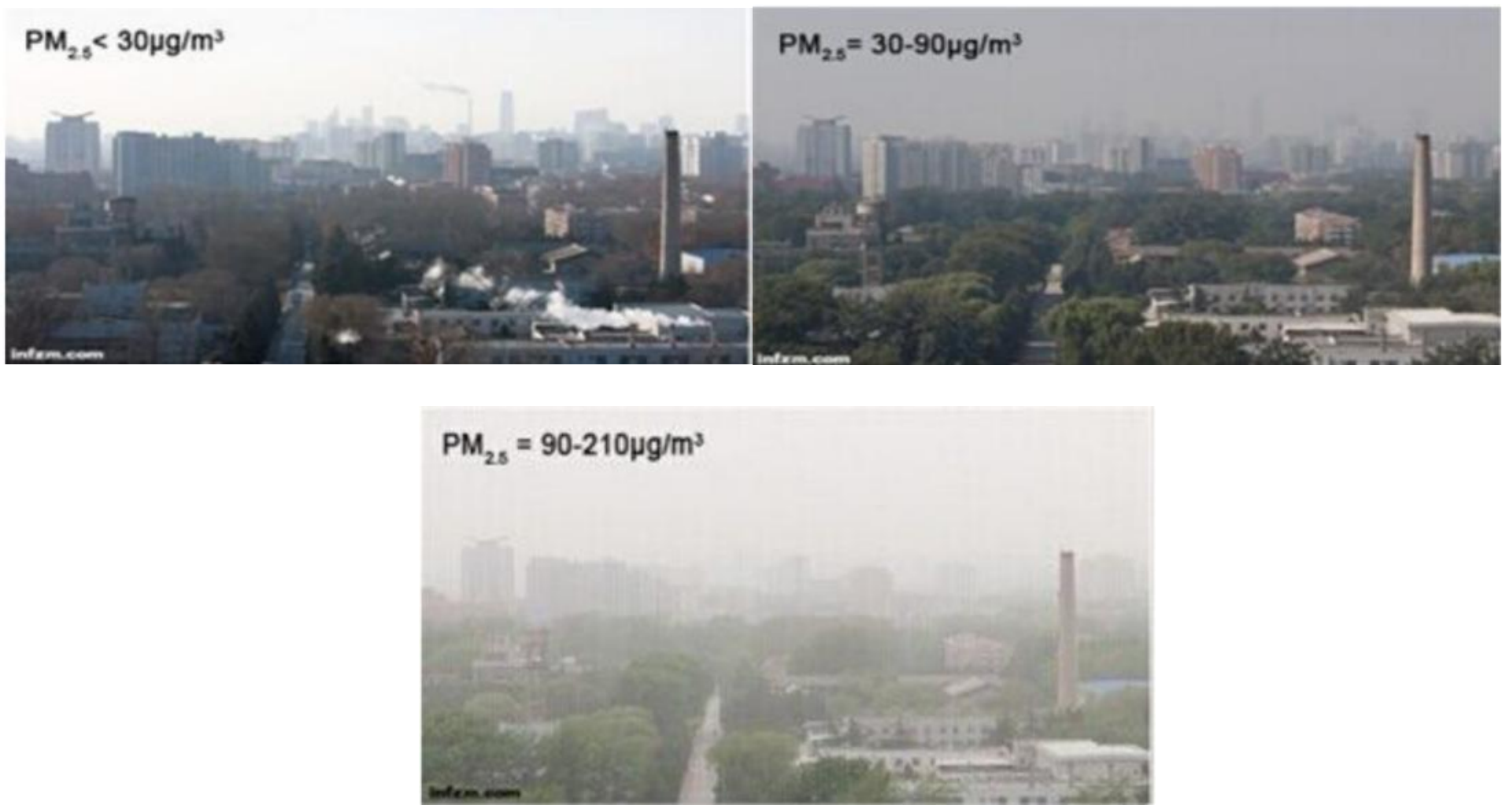

Annual Average PM2.5 Concentrations in Major Chinese Cities (2016):
Haikou: 26 μg/m³ | Shanghai: 61 μg/m³ | Wuhan: 89 μg/m³ | Xi'an: 104 μg/m³ | Shijiazhuang: 148 μg/m³

If, after the government implements pollution prevention and control actions, the annual average PM2.5 concentration in your city reaches the following levels within the next three years (compared to Nanjing's 2017 level of 86.3 μg/m³), how acceptable would it be to you?

	Completely Acceptable	Readily Acceptable	Moderately Acceptable	Somewhat Unacceptable	Unacceptable
PM2.5 annual average concentration reduction: 0%	○	○	○	○	○
PM2.5 annual average concentration reduction: 10%	○	○	○	○	○
PM2.5 annual average concentration reduction: 20%	○	○	○	○	○
PM2.5 annual average concentration reduction: 40%	○	○	○	○	○
PM2.5 annual average concentration reduction: 50%	○	○	○	○	○
PM2.5 annual average concentration reduction: 65%	○	○	○	○	○
PM2.5 annual average concentration reduction: 80%	○	○	○	○	○
PM2.5 annual average concentration reduction: 85%	○	○	○	○	○

Part III

Please record your detailed activities over the past 24 hours.

	Home	Office	Public transport	Private car/ Taxi	Walking / Cycling	Other indoor activities	Other outdoor activities	Going out for COVID test
6:00-7:00	○	○	○	○	○	○	○	○
7:00-8:00	○	○	○	○	○	○	○	○
8:00-9:00	○	○	○	○	○	○	○	○
9:00-10:00	○	○	○	○	○	○	○	○
10:00-11:00	○	○	○	○	○	○	○	○
11:00-12:00	○	○	○	○	○	○	○	○
12:00-13:00	○	○	○	○	○	○	○	○
13:00-14:00	○	○	○	○	○	○	○	○
14:00-15:00	○	○	○	○	○	○	○	○
15:00-16:00	○	○	○	○	○	○	○	○
16:00-17:00	○	○	○	○	○	○	○	○
17:00-18:00	○	○	○	○	○	○	○	○
18:00-19:00	○	○	○	○	○	○	○	○
19:00-20:00	○	○	○	○	○	○	○	○
20:00-21:00	○	○	○	○	○	○	○	○
21:00-22:00	○	○	○	○	○	○	○	○
22:00-23:00	○	○	○	○	○	○	○	○
23:00-0:00	○	○	○	○	○	○	○	○
0:00-1:00	○	○	○	○	○	○	○	○
1:00-2:00	○	○	○	○	○	○	○	○
2:00-3:00	○	○	○	○	○	○	○	○
3:00-4:00	○	○	○	○	○	○	○	○
4:00-5:00	○	○	○	○	○	○	○	○
5:00-6:00	○	○	○	○	○	○	○	○

Part IV

Personal Information

1. Gender: [Single choice]

○ Male ○ Female

2. Age: _______ years [Fill-in-the-blank]

3. Weight: _____ kg; Height: _____ cm [Fill-in-the-blank]

7. Highest Education Level: [Single choice]

○ No formal education ○ Primary school ○ Junior high school ○ High school/Vocational school

○ College/Associate degree ○ Master's degree or higher

8. Occupation: (Retirees: state former occupation) [Single choice]

○ Civil servant ○ Public institution staff ○ Enterprise employee ○ Self-employed ○ Farmer ○ Homemaker ○ Student

○ Freelancer ○ Other

8.1 Public Institution Type: [Single choice]

○ Education/Science/Culture/Sports ○ Healthcare ○ Social welfare ○ Transportation ○ Urban utilities

○ Agriculture/Forestry/Fishing ○ Geoscience & Environmental Protection ○ Other

9. Monthly Household Income Per Capita: [Single choice]

○ < ¥1,000 ○ ¥1,000–2,000 ○ ¥2,000–4,000 ○ ¥4,000–8,000 ○ ¥8,000–20,000

○ ¥20,000–40,000 ○ ¥40,000–80,000 ○ ¥80,000–120,000 ○ ¥120,000–200,000 ○ > ¥200,000

10. Residential District in Nanjing, Jiangsu: [Single choice]

○ Gulou ○ Xuanwu ○ Qixia ○ Qinhuai ○ Jiangning ○ Pukou ○ Jianye ○ Yuhuatai

11. During the pandemic, which type of mask did you typically use?

○ Cloth mask ○ Standard medical mask ○ Surgical mask ○ N95/KN95

12. During the pandemic, in which situations did you consistently wear masks? [Multiple choice]

□ At home/indoor activities □ Outdoor activities (including COVID testing) □ Using public transport

□ Driving/riding in private cars □ Taking taxis □ Walking □ Cycling

13. During the pandemic, your average daily mask-wearing time was ______ minutes.

14. Based on personal experience, your physiological reactions to air pollution include:[Multiple choice]

□ Cough □ Chest tightness/difficulty breathing □ Headache/dizziness □ Throat discomfort (soreness, hoarseness)

□ Allergies □ Lethargy □ Skin itching □ Anxiety due to worry □ Irritability □ Dry/watery eyes

□ Nasal congestion/runny nose □ Other symptoms

Living environment:

	In 20 m	20~100 m	100~500 m	0.5~1 km	Above 1 km
The nearest major transportation artery	○	○	○	○	○
The nearest active construction site	○	○	○	○	○
The nearest factory potentially emitting air pollutants	○	○	○	○	○

Text S3. Questionnaire on Nanjing public perception of air pollution.

Maximum acceptable risk level: In the PRAR assessment, participants were shown visual simulations of different levels of $PM_{2.5}$. Respondents were asked to rate their acceptance of the $PM_{2.5}$ concentrations in the Action Plan on five levels: "Completely Acceptable", "Easily Acceptable", "Basically Acceptable", "Difficult to Acceptable", and "Not Acceptable". 2017 is the base year for our scenarios. The $PM_{2.5}$ concentration reduction rates of the scenarios were 85% (10.05 mg/m^3), 80% (13.40 mg/m^3), 65% (23.45 mg/m^3), 50% (33.50 mg/m^3), 40% (40.20 mg/m^3), 20% (53.60 mg/m^3), and 10% (60.30 mg/m^3). The remaining percentage was multiplied by the average $PM_{2.5}$ concentration level in 2017, and the $PM_{2.5}$ reduction percentage was converted into $PM_{2.5}$ concentration level using the following formula (1), where C_d is the $PM_{2.5}$ concentration (mg/m^3); C_0 is the average $PM_{2.5}$ concentration in 2017, which is 86.3 mg/m^3.

$$C_d = C_0 \times (100\% - decrease\%) \quad (1)$$

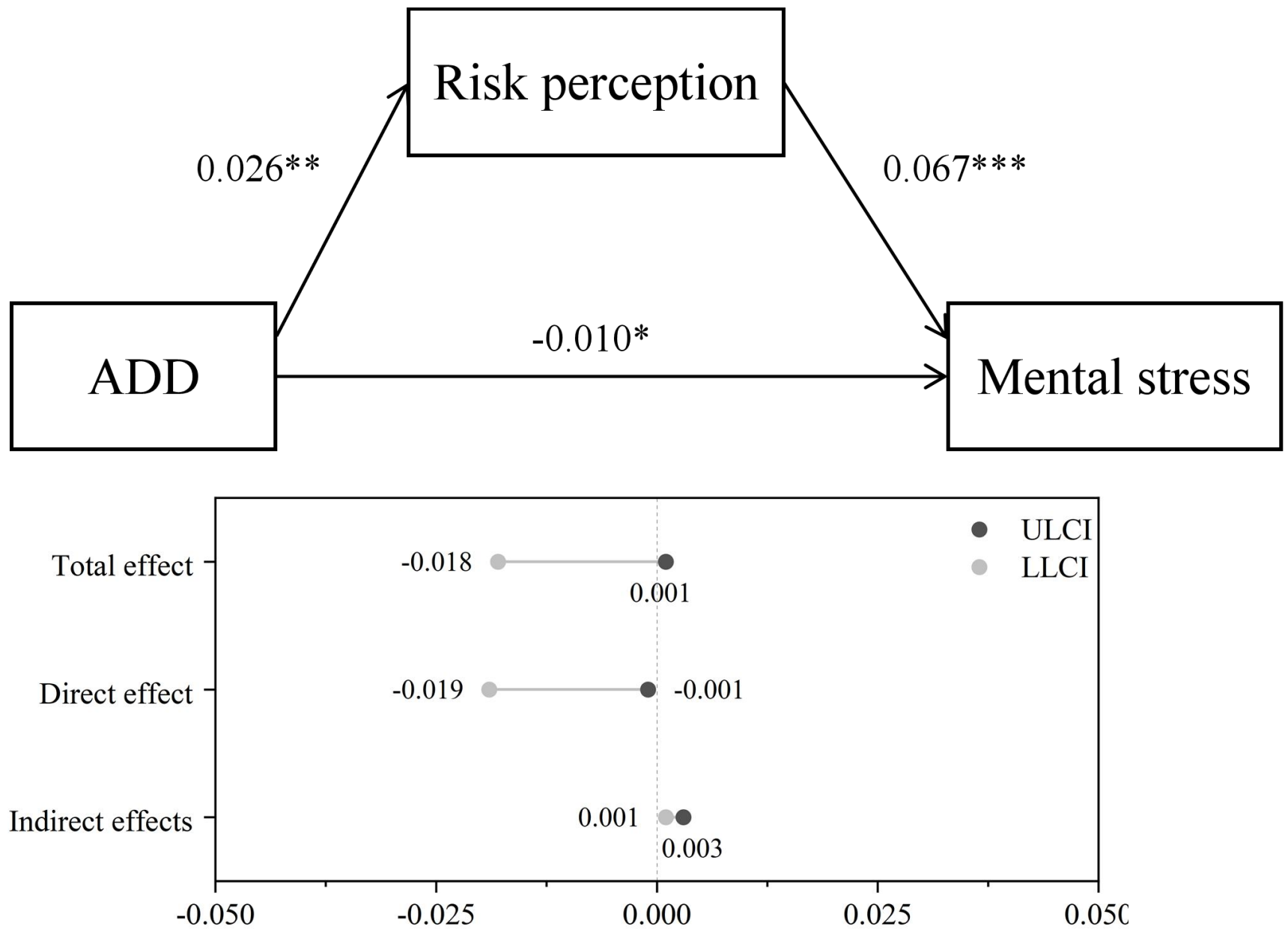

Fig. S1. Path analysis of the impact of ADD on mental stress. *p<0.05, **p<0.01, ***p<0.001.

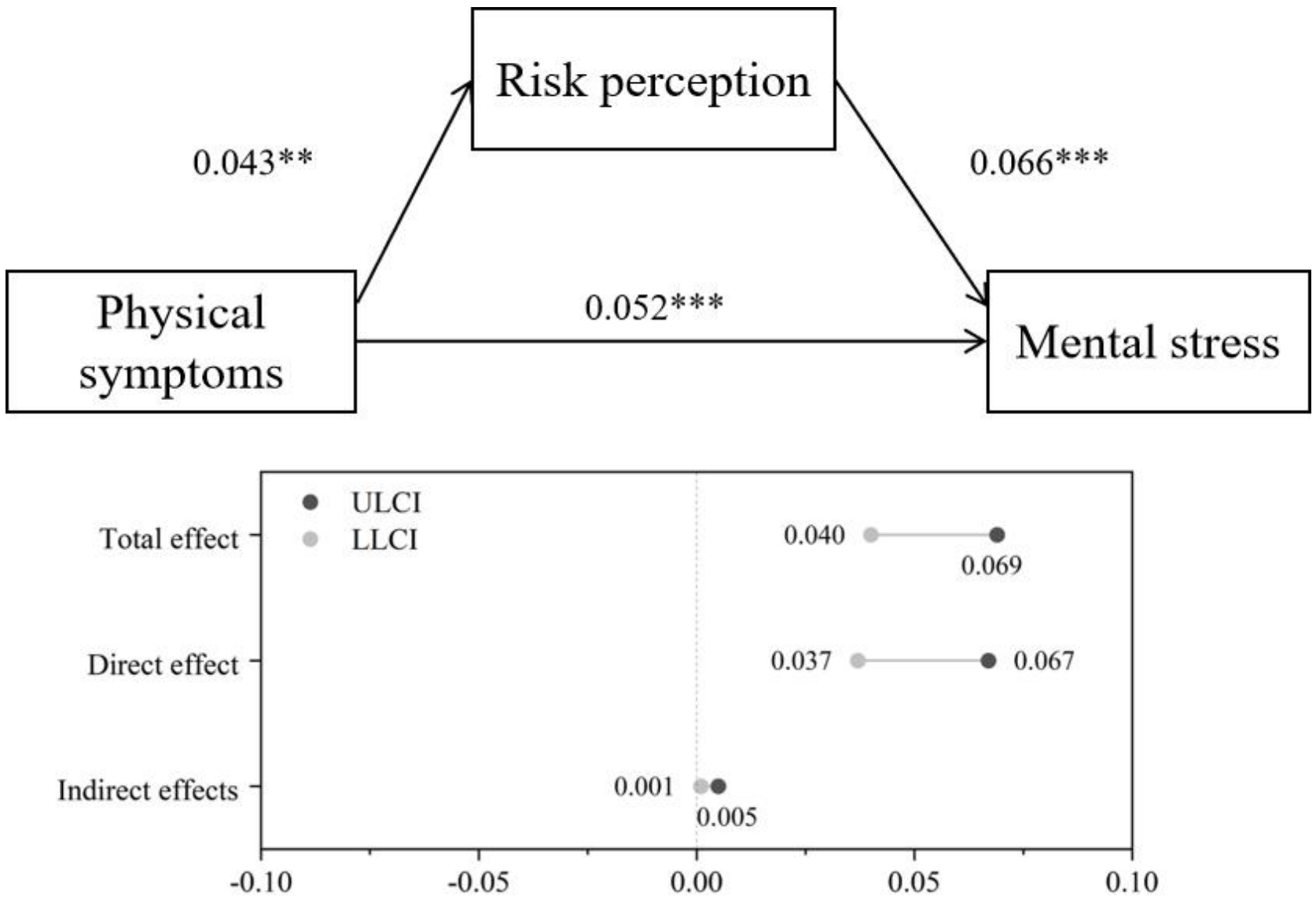

Fig. S2. Path analysis of the impact of physical symptoms on mental stress. *p<0.05, **p<0.01, ***p<0.001.

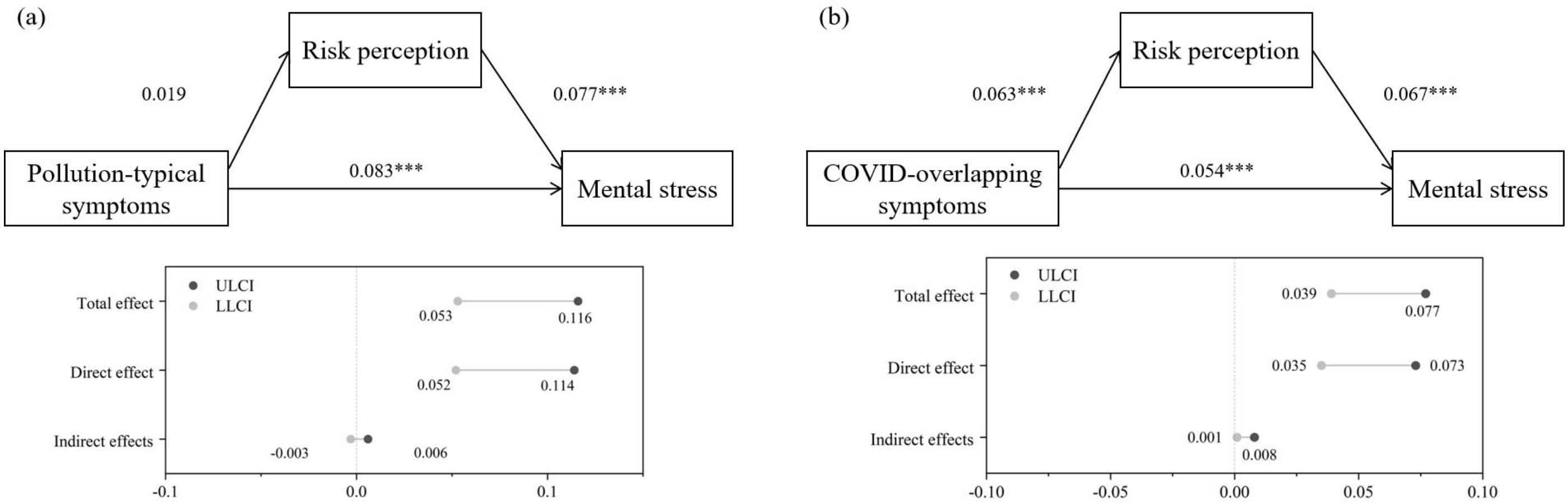

Fig. S3. Path analysis of the impact of (a) pollution-typical irritation symptoms and (b) COVID-overlapping symptoms on mental stress. *p<0.05, **p<0.01, ***p<0.001.

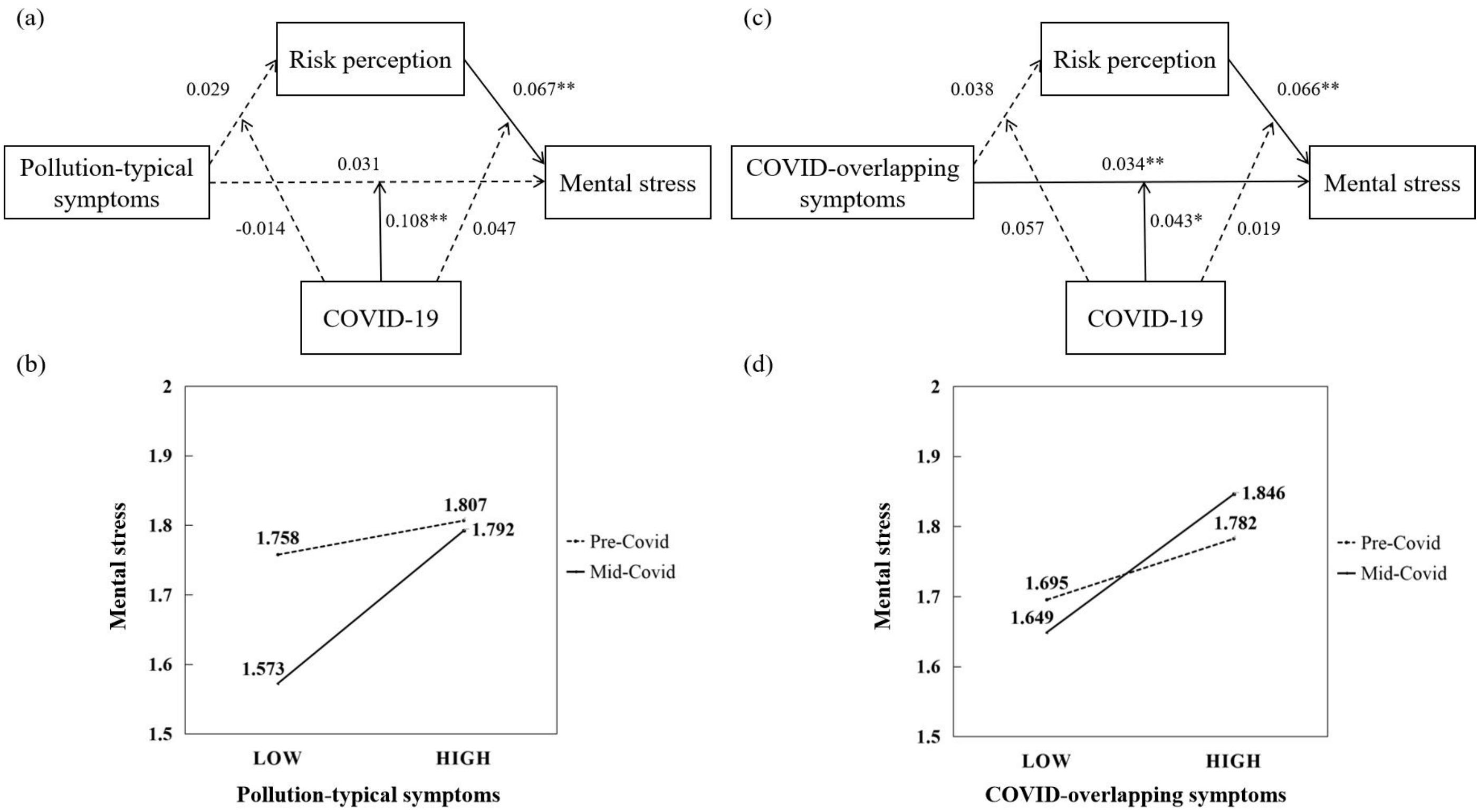

Fig. S4. Sensitivity analysis: COVID-19 crisis amplified the impact of health threats on mental health. (a) pathway of Model III (pollution-typical irritation symptoms), (b) Moderating effect of COVID-19 (pollution-typical irritation symptoms), (c) pathway of Model III (COVID-overlapping symptoms), (d) Moderating effect of COVID-19 (COVID-overlapping symptoms) .*p<0.05, **p<0.01, ***p<0.001.

Table S1. Comparison of variable means before and after the COVID-19 pandemic.

	Pre-Covid	Mid- Covid	Levene's Test (p)	Independent Samples t-test (p)	Mann-Whitney U（p）
Familiarity	3.417	3.754	0.954	<0.001	—
Risk	3.884	3.805	0.010	—	0.033
Trust in government	2.978	3.707	0.381	<0.001	—
ADD	8.569	4.027	<0.001	—	<0.001
Physical Symptoms	3.236	3.156	0.001	—	0.941
Mental Stress	1.639	1.673	<0.001	—	0.281

Table S2. Multiple linear stepwise regression results for Model I.

Variables	ADD		Trust in government		Risk perception		
	(1)	(2)	(3)	(4)	(5)	(6)	(7)
Gender	0.021	0.030	0.016	0.012	0.048	0.050	0.050
Age	-0.071*	-0.189***	-0.088**	-0.035	0.002	-0.026	-0.031
Education	0.100**	-0.078**	-0.096**	-0.018	0.031	-0.010	-0.012
Income	-0.408***	-0.095***	0.208***	0.069*	-0.020	0.053	0.046
Physical symptoms	-0.015	-0.040*	-0.076**	-0.065*	0.105***	0.099**	0.101***
Living environment	-0.107***	0.001	0.034	-0.014	-0.027	-0.002	-0.001
Familiarity	-0.070*	0.054**	0.365***	0.310***	0.402***	0.431***	0.416***
COVID-19		-0.737***		0.326***		-0.172***	-0.219***
ADD							-0.038
Trust in government							0.057
R^2	0.216	0.617	0.201	0.279	0.183	0.205	0.207
ΔR^2	0.216	0.401	0.201	0.078	0.183	0.022	0.003
F	39.414***	200.979***	35.924***	48.391***	31.955***	32.130***	26.093***
ΔF	39.414***	1044.139***	35.924***	108.603***	31.955***	27.440***	1.752

*p < 0.05, **p < 0.01, ***p < 0.001.

Table S3. Changes between 2019 and 2021 in the perception of respondents identifying *Health*, *Environment*, and *Pollution* as the greatest source of risk in their daily lives.

Countries	Year	Health	Environment	Pollution
Total	2019	14.67%	3.44%	0.62%
	2021	19.57%	3.13%	0.38%
	Pearson χ^2	1189.538***	20.435***	80.773***
Low-income	2019	14.58%	3.67%	0.28%
	2021	14.35%	2.75%	0.30%
	Pearson χ^2	0.262	15.154***	0.103
Lower-middle-income	2019	12.42%	3.72%	0.37%
	2021	20.89%	3.76%	0.27%
	Pearson χ^2	983.597***	0.074	5.425*
Upper-middle-income	2019	15.48%	3.33%	0.59%
	2021	16.67%	2.11%	0.34%
	Pearson χ^2	22.105***	116.604***	26.105***
High-income	2019	15.72%	3.20%	1.03%
	2021	22.24%	3.55%	0.51%
	Pearson χ^2	602.079***	8.317**	76.224***

*$p < 0.05$, **$p < 0.01$, ***$p < 0.001$.

Table S4. Multiple linear stepwise regression results for Model II.

Variables	Risk perception		Mental stress		
	(1)	(2)	(3)	(4)	(5)
Gender	0.048	0.049	0.065*	0.064*	0.058
Age	0.002	-0.024	-0.128***	-0.118***	-0.115**
Education	0.031	-0.009	-0.084*	-0.068	-0.067
Income	-0.019	0.050	0.046	0.018	0.012
Physical symptoms	0.105***	0.103***	0.243***	0.243***	0.230***
Living environment	-0.027	-0.001	0.016	0.006	0.006
Familiarity	0.403***	0.414***	-0.005	-0.009	-0.063
Trust in government	-0.001	0.056	-0.079*	-0.102**	-0.110**
COVID-19		-0.190***		0.078*	0.102**
Risk perception					0.131***
R^2	0.183	0.207	0.088	0.092	0.105
ΔR^2	0.183	0.024	0.088	0.004	0.014
F	27.933***	28.926***	12.001***	11.191***	11.730***
ΔF	27.933***	30.312***	12.001***	4.386*	15.155***

*p < 0.05, **p < 0.01, ***p < 0.001.

Table S5. The direct effects of three categories of physical symptoms under different COVID-19 conditions.

**Physical symptoms**						
COVID-19	Effect	se	t	p	LLCI	ULCI
Pre-Covid	0.027	0.010	2.715	0.007	0.008	0.047
Mid-Covid	0.081	0.011	7.173	0.000	0.059	0.103
**Pollution-typical symptoms**						
COVID-19	Effect	se	t	p	LLCI	ULCI
Pre-Covid	0.031	0.022	1.419	0.156	-0.012	0.073
Mid-Covid	0.138	0.022	6.156	0.000	0.094	0.182
**COVID-overlapping symptoms**						
COVID-19	Effect	se	t	p	LLCI	ULCI
Pre-Covid	0.034	0.013	2.602	0.009	0.008	0.059
Mid-Covid	0.077	0.015	5.179	0.000	0.048	0.106

Table S6. Risk perception scale measurement equivalence.

Model	CFI	TLI	RMSEA	SRMR	ΔCFI	ΔRMSEA
Configural	0.992	0.989	0.0338	0.0622		
Metric	0.991	0.989	0.034	0.0633	-0.000684	0.000153
Scalar	0.994	0.992	0.0295	0.058	0.0021	-0.00445

Notes:

1. Multi-group CFA conducted across Pre-Covid vs Mid-Covid samples using WLSMV estimator and theta parameterization for ordered indicators.
2. Invariance was evaluated sequentially (configural → metric/loadings → scalar/loadings+thresholds).
3. Changes in fit indices indicate adequate invariance (e.g., |ΔCFI| < 0.01; ΔRMSEA < 0.015).

Table S7. Raking calibration weights.

Variable	Unweighted	Weighted
Gender	0.049	-0.004
Age	-0.024	0.004
Education	-0.009	-0.027
Income	0.05	0.054
Physical symptoms	0.103***	0.079***
Living environment	-0.001	0.067
Familiarity	0.414***	0.450***
Trust in government	0.056	0.062
COVID-19	-0.190***	-0.441***

Notes: Raking (iterative proportional fitting) weights were constructed to calibrate the sample marginals of gender, age group, and education to Nanjing's citywide population margins (2020 census statistics). Weights were generated separately for each wave, normalized within wave (mean = 1), and winsorized at the 99th percentile. Weighted estimates were obtained using design-based standard errors. *p < 0.05, **p < 0.01, ***p < 0.001.

Table S8. Inter-coder reliability (human validation).

Task (binary)	N posts	Agreement (%)	Cohen's κ	a (Yes/Yes)	b (Yes/No)	c (Yes/No)	d (No/No)
COVID/Air-pollution-related (Yes/No)	300	97.00	0.524	286	4	5	5

Notes:

1. The table reports inter-coder reliability based on a random sample of N = 300 posts independently labeled by two human coders.
2. Agreement (%) is the proportion of posts on which the two coders assigned the same label:

$(a + b) / N$

3. Cohen's κ corrects raw agreement for chance agreement based on marginal label distributions:

$\kappa = (P_0 - P_e)/(1 - P_e)$, where $P_0 = (a + b) / N$, and $P_e = [(a + b) / N \cdot (a + b) / N] + [(c + d) / N \cdot (b + d) / N]$.

4. Because the "Yes" label is highly prevalent in this sample (class imbalance), κ may be lower than the raw agreement would suggest; therefore, we report both Agreement (%) and κ for transparency.
5. Coding was performed using the same binary definitions as in the main text (COVID/Air-pollution-related: Yes/No).

Table S9. Sensitivity analysis using overlapping countries in the World Risk Poll (2019 vs 2021), stratified by World Bank income groups.

Health risk							
Income group	Full sample 2019	Full sample 2021	Full sample χ^2	Common countries 2019	Common countries 2021	Common countries χ^2	Pattern consistency
Low income	14.58%	14.35%	0.262	15.30%	14.30%	3.472	Yes
Lower-middle income	12.42%	20.89%	983.597***	12.50%	20.90%	905.151***	Yes
Upper-middle income	15.48%	16.67%	22.105***	15.00%	16.80%	44.217***	Yes
High income	15.72%	22.24%	602.079***	16.20%	22.50%	532.719***	Yes
All countries	14.67%	19.57%	1189.538***	14.80%	19.70%	1076.958***	Yes
Broad environmental risk (including pollution)							
Income group	Full sample 2019	Full sample 2021	Full sample χ^2	Common countries 2019	Common countries 2021	Common countries χ^2	Pattern consistency
Low income	3.67%	2.75%	15.154***	4.70%	2.70%	48.312***	Yes
Lower-middle income	3.72%	3.76%	0.074	3.70%	3.80%	0.203	Yes
Upper-middle income	3.33%	2.11%	116.604***	3.30%	2.10%	106.091***	Yes
High income	3.20%	3.55%	8.317**	3.20%	3.60%	10.23**	Yes
All countries	3.44%	3.13%	20.435***	3.50%	3.10%	26.404***	Yes
Narrow pollution							
Income group	Full sample 2019	Full sample 2021	Full sample χ^2	Common countries 2019	Common countries 2021	Common countries χ^2	Pattern consistency
Low income	0.28%	0.30%	0.103	0.40%	0.30%	0.383	Mostly
Lower-middle income	0.37%	0.27%	5.425*	0.30%	0.30%	2.306	Mostly
Upper-middle income	0.59%	0.34%	26.105***	0.60%	0.30%	24.438***	Yes
High income	1.03%	0.51%	76.224***	1.00%	0.50%	61.876***	Yes
All countries	0.62%	0.38%	80.773***	0.60%	0.40%	81.281***	Yes

Notes:

1. Percentages indicate the share of respondents selecting [define the outcome clearly here: e.g., “health risk” / “narrow pollution” / “broad environmental risk”] as the greatest risk to safety in daily life.
2. “Full sample” uses all countries available in each wave (2019: 139 countries; 2021: 121 countries). “Common countries” restricts analyses to countries appearing in both waves.
3. Chi-square tests compare proportions between 2019 and 2021 within each income group.
4. “Pattern consistency” indicates whether the direction and substantive magnitude of change in the common-country sample is consistent with the full-sample result (e.g., Yes / Mostly / No).
5. *$p < 0.05$, **$p < 0.01$, ***$p < 0.001$.

Table S10. Regression analysis results of air pollution risk perception on government trust.

Independent variables \ Dependent variable	Risk perception	
Gender	0.048	0.048
Age	0.002	0.002
Education	0.031	0.031
Income	-0.02	-0.019
Physical symptoms	0.105***	0.105***
Living environment	-0.027	-0.027
Familiarity	0.402***	0.403***
Trust	-	-0.001
R^2	0.183	0.183
ΔR^2	0.183	0.000
F	31.955***	27.933***
ΔF	31.955***	0.002

*p < 0.05, **p < 0.01, ***p < 0.001.

Table S11. Sample distribution statistics by region.

Region	Sample Size（N=508+500=1008）	Population
Gaochun District	10(0.99%)	429173(4.60%)
Gulou District	161(15.97%)	940387(10.10%)
Jianye District	99(9.82%)	534257(5.74%)
Jiangning District	139(13.79%)	1926117(20.68%)
Lishui District	15(1.49%)	491336(5.27%)
Luhe District	19(1.88%)	946563(10.16%)
Pukou District	109(10.81%)	1171603(12.58%)
Qixia District	107(10.62%)	987835(10.61%)
Qinhuai District	139(13.79%)	740809(7.95%)
Xuanwu District	138(13.69%)	537825(5.77%)
Yuhuatai District	72(7.14%)	608780(6.54%)

Table S12. Demographic characteristics.

	Characteristic	Count	Category	Standard Deviation
Pre-pandemic (2018-2019)	Gender	261	Male	0.50
		247	Female	
	Age	99	≤20	1.31
		191	21-29	
		101	30-39	
		66	40-49	
		32	50-60	
		19	>60	
	Highest Education	25	Junior HS or below	0.65
		52	High school (includes vocational high school)	
		365	College/University (includes associate degree programs)	
		66	Postgraduate and higher degrees	
	Monthly Income (¥)	100	<5000	1.13
		320	5000-10000	
		82	>10000	
During Pandem-ic (2022)	Gender	250	Male	0.50
		250	Female	
	Age	98	≤20	1.28
		188	21-29	
		110	30-39	
		60	40-49	
		26	50-60	
		18	>60	
	Highest Education	34	Junior HS or below	0.64
		49	High school (includes vocational high school)	
		383	College/University (includes associate degree programs)	
		34	Postgraduate and higher degrees	
	Monthly Income (¥)	165	<5000	1.32
		241	5000-10000	
		94	>10000	

Table S13. Demographic characteristics (residential environment metrics).

Residential Environment Metrics	Number of People	Strata	Standard Deviation
Distance from Residence/Workplace to Major Roads	585	<500 m	1.171
	233	500-1000 m	
	190	>1000 m	
Distance from Residence/Workplace to Construction Sites	281	<500 m	1.082
	232	500-1000 m	
	495	>1000 m	
Distance from Residence/Workplace to Potentially Polluting Factories	71	<500 m	0.642
	100	500-1000 m	
	837	>1000 m	

Table S14. Factor analysis loading matrix (perceptions of air pollutioin).

Mid-Covid				
Items	Trust	Familiarity	Risk	Control
11	0.820	0.213	0.135	0.098
12	0.816	0.087	0.175	0.162
10	0.743	0.220	0.118	0.121
7	0.528	0.405	-0.069	0.262
2	0.202	0.751	0.327	0.100
1	0.220	0.715	0.367	0.076
3	0.233	0.712	0.100	0.130
6	0.200	0.083	0.822	0.050
4	0.079	0.234	0.812	0.097
5	0.048	0.419	0.659	0.236
9	0.103	0.054	0.067	0.819
8	-0.018	-0.002	0.027	0.770
Explained variance	20.70%	18.47%	17.75%	11.50%
Cumulative variance	20.70%	39.17%	56.92%	68.42%
Pre-Covid				
Items	Risk	Trust	Familiarity	Control
5	0.838	0.052	0.077	0.120
4	0.823	0.016	0.140	0.155
6	0.757	-0.054	0.138	0.026
11	0.121	0.831	-0.043	-0.039
10	-0.097	0.754	0.062	0.113
12	0.102	0.688	0.073	0.151
7	-0.137	0.520	0.284	0.029
3	-0.043	0.149	0.759	0.128
2	0.210	0.096	0.748	0.155
1	0.286	0.010	0.712	-0.075
9	0.060	0.189	-0.004	0.828
8	0.198	0.016	0.188	0.766
Explained variance	18.13%	17.28%	15.13%	11.64%
Cumulative variance	18.13%	35.41%	50.54%	62.18%

Table S15. KMO and Bartlett's test.

	Pre-pandemic (2018-2019)				During pandemic (2022)			
	KMO	Bartlett's sphericity test			KMO	Bartlett's sphericity test		
		χ^2	Degree of freedom	Significance		χ^2	Degree of freedom	Significance
Perceived Stress Scale	0.737	624.98	66	<0.001	0.878	2219.12	66	0.000
Mental Stress Score	0.731	213.06	10	<0.001	0.796	529.73	10	<0.001

Table S16. Cronbach's alpha of perception scales (perceptions of air pollutioin).

Perception variables	Cronbach α
**Mid-Covid**	
Familiarity	0.776
Risk	0.770
Control	0.535
Trust	0.806
**Pre-Covid**	
Familiarity	0.657
Risk	0.775
Control	0.396
Trust	0.686

Table S17. Confirmatory factor analysis results (validity tests).

Phase	CMIN	DF	CMIN/DF	CFI	TLI
Pre-Covid	50.744	24	2.114	0.974	0.961
Mid-Covid	53.346	24	2.223	0.983	0.975

Table S18. Cronbach's alpha of perception scales (mental stress).

	**Items**	Cronbach's Alpha	
		Pre-pandemic (2018-2019)	Mid-pandemic (2022)
Psychological Stress Score	5	0.605	0.752

Table S19. Variance Inflation Factor (VIF).

Variables	ADD		Risk perception		
	**Model 1**	**Model 2**	**Model 3**	**Model 4**	**Model 5**
Gender	1.010	1.010	1.010	1.010	1.013
Age	1.203	1.231	1.203	1.231	1.323
Education	1.253	1.320	1.253	1.320	1.336
Income	1.207	1.405	1.207	1.405	1.430
Physical symptoms	1.021	1.021	1.021	1.021	1.024
Living environment	1.025	1.053	1.025	1.053	1.053
Familiarity	1.225	1.230	1.225	1.230	1.235
Trust in government	1.251	1.388	1.251	1.388	1.392
COVID-19		1.505		1.505	2.979
ADD					2.618
**Variables**	**ADD**		**Trust**		
	**Model 6**	**Model 7**	**Model 8**	**Model 9**	**Model 10**
Gender	1.013	1.013	1.013	1.013	1.016
Age	1.194	1.230	1.194	1.230	1.324
Education	1.243	1.320	1.243	1.320	1.336
Income	1.154	1.402	1.154	1.402	1.425
Physical symptoms	1.027	1.027	1.027	1.027	1.031
Living environment	1.024	1.052	1.024	1.052	1.052
Familiarity	1.257	1.331	1.257	1.331	1.341
Risk	1.224	1.257	1.224	1.257	1.258
COVID-19		1.395		1.395	2.825
ADD					2.611
**Variables**	**Risk perception**		**Mental stress**		
	**Model 11**	**Model 12**	**Model 13**	**Model 14**	**Model 15**
Gender	1.010	1.010	1.010	1.010	1.013
Age	1.203	1.231	1.203	1.231	1.231
Education	1.253	1.320	1.253	1.320	1.320
Income	1.207	1.405	1.207	1.405	1.408
Physical symptoms	1.021	1.021	1.021	1.021	1.034
Living environment	1.025	1.053	1.025	1.053	1.053
Familiarity	1.225	1.230	1.225	1.230	1.446
Trust in government	1.251	1.388	1.251	1.388	1.391
COVID-19		1.505		1.505	1.551
Risk					1.261
